# Transcriptome analysis provides insight into the regulatory mechanisms underlying pollen germination recovery at normal high ambient temperature in wild banana (*Musa itinerans*)

**DOI:** 10.3389/fpls.2023.1255418

**Published:** 2023-09-26

**Authors:** Chunyu Zhang, Chengyu Zhang, Xiaoqiong Xu, Minzhang Liao, Ning Tong, Zihao Zhang, Yukun Chen, Xu Xu Han, Yuling Lin, Zhongxiong Lai

**Affiliations:** ^1^ Institute of Horticultural Biotechnology, Fujian Agriculture and Forestry University, Fuzhou, China; ^2^ Institut de la Recherche Interdisciplinaire de Toulouse, IRIT-ARI, Toulouse, France

**Keywords:** wild banana, RNA-seq, pollen fertility, germination recovery, sugar metabolism, pollen wall

## Abstract

**Introduction:**

Cultivated banana are polyploid, with low pollen fertility, and most cultivars are male sterile, which leads to difficulties in banana breeding research. The selection of male parent with excellent resistance and pollen fertility is therefore essential for banana breeding. Wild banana (*Musa itinerans*) have developed many good characteristics during natural selection and constitute an excellent gene pool for breeding. Therefore, research on wild banana breeding is very important for banana breeding.

**Results:**

In the current analysis, we examined the changes in viability of wild banana pollens at different temperatures by *in vitro* germination, and found that the germination ability of wild banana pollens cultured at 28°C for 2 days was higher than that of pollens cultured at 23°C (pollens that could not germinate normally under low temperature stress), 24°C (cultured at a constant temperature for 2 days) and 32°C (cultured at a constant temperature for 2 days). To elucidate the molecular mechanisms underlying the germination restoration process in wild banana pollens, we selected the wild banana pollens that had lost its germination ability under low temperature stress (23°C) as the control group (CK) and the wild banana pollens that had recovered its germination ability under constant temperature incubation of 28°C for 2 days as the treatment group (T) for transcriptome sequencing. A total of 921 differentially expressed genes (DEGs) were detected in CK vs T, of which 265 were up-regulated and 656 were down-regulated. The combined analysis of Gene Ontology (GO) and Kyoto Encyclopedia of Genes and Genomes (KEGG) revealed that the activation, metabolism of various substances (lipids, sugars, amino acids) play a major role in restoring pollen germination capacity. TCA cycle and the sesquiterpenoid and triterpenoid biosynthetic pathways were also significantly enriched in the KEGG pathway. And we found that some DEGs may be associated with pollen wall formation, DNA methylation and DNA repair. The cysteine content, free fatty acid (FFA) content, H_2_O_2_ content, fructose content, and sucrose content of pollen were increased at treatment of 28°C, while D-Golactose content was decreased. Finally, the GO pathway was enriched for a total of 24 DEGs related to pollen germination, of which 16 DEGs received targeted regulation by 14 MYBs.

**Discussions:**

Our study suggests that the balance between various metabolic processes, pollen wall remodelling, DNA methylation, DNA repairs and regulation of MYBs are essential for germination of wild banana pollens.

## 1 Introduction

Bananas (*Musa nana* Lour.) are tropical and subtropical fruit trees that are valued for their unique flavour. However, its yield and quality have been threatened by natural disasters and various diseases during cultivation, and the selection of a number of resistant bananas has become a major breeding direction. As a relatively controlled and stable breeding method, crossing presupposes the availability of male parent. However, cultivated bananas (*Musa* spp.) are derived from intraspecific or interspecific crosses between diploid wild species (*Musa acuminata*) and (*Musa balbisiana*) and are mainly triploid, with three genotypes, AAA, AAB and ABB. Cultivated bananas produce fruit mainly by unisexual fruiting, with very few or no seeds, so that most cultivated banana varieties are highly sterile in both sexes (Pillay and Tripathi, 2007; Soares et al., 2014), which leads to difficulties in banana breeding research. Wild bananas (*Musa itinerans*) have developed many interesting characteristics during long-term natural selection, such as resistance to cold, pests and diseases. In addition, wild bananas are diploid and have fertile seeds, which makes them a good gene library for genetic improvement of cultivated bananas. Therefore, research on wild banana breeding is very important for banana breeding. There were abundant wild banana resources in Fujian, and much progress in wild banana resistance research has made (Liu et al., 2018), but there is still a gap in wild banana pollen breeding research.

The development of plant pollen is essential for the sexual reproduction of seed plants and for the alternation of generations. It is a finely regulated but complex and fragile process, and any error in this process can lead to male sterility. Many studies have shown that temperature changes have a significant impact on pollen fertility. The main effects of heat stress on pollen development are degradation of the early felt layer, involuntary anther weight, deformation of pollen grains, impaired pollination and possible thickening of the pollen wall (Jiang et al., 2015), as well as accumulation of ROS, which inhibits pollen tube growth (Muhlemann et al., 2018). The HT-sensitive cotton line H05 showed *GhAOC2* expression in anthers under high temperature treatment, which reduced JA biosynthesis and led to excessive ROS accumulation in anthers, resulting in male sterility (Khan et al., 2023). Studies have shown that this increase in ROS content may be closely related to a decrease in the activity of several antioxidant enzymes (Zhao et al., 2018). In addition, a variety of substances may also provide protection against pollen development in response to high temperatures, for example, sorbitol synthesis is important for pollen growth and development, and in apples, reduced sorbitol synthesis inhibits MYB39L expression, leading to abnormal pollen development and reduced pollen tube growth (Meng et al., 2018). Melatonin mitigates high temperature-induced male sterility by upregulating the transcription and activity of several antioxidant enzymes (Qi et al., 2018). Similarly, the effect of low temperature on pollen fertility is significant, and treatment of rice pollens at low temperature can delay the degradation of the innermost layer of the anther wall, the tapetum, by altering the insoluble polysaccharide and protein levels in the anther wall (Tian et al., 2015), and also lead to premature callose (1,3-glucan) breakdown and lack of normal pollen wall formation (Mamun et al., 2013). TMS10-like redundantly controls male fertility with TMS10, tms10 at high temperature in rice. The tms10 mutant shows male sterility at high temperature, while the opposite is true at low temperature. The double mutants tms10 and tms10l show pollen sterility at both high and low temperatures (Yu et al., 2017). Low temperatures also affect a range of metabolic processes in the plant that it cause pollen sterility in cold-sensitive chickpea varieties, mainly due to disruption of starch and proline metabolism in the anthers (Kiran et al., 2021) and affect the normal metabolism of sugars during spore development in rice (Mamun et al., 2013). In general, pollens are very sensitive to temperature changes during its development, the right temperature is very important for pollen growth and development.

Transcriptomes of many species have revealed fertility mechanisms, for example chickpea (Kiran et al., 2021), rice (Li et al., 2020), maize (Han et al., 2022; Nelms and Walbot, 2022), cotton (Ma et al., 2021) and tobacco (Krawczyk et al., 2022). In addition, some species have also sequenced transcriptome by knocking out a gene, thus revealing the regulatory network during pollen germination (Chen et al., 2018). Carbohydrates and their derivatives have also been found to regulate pollen viability based on RNA-Seq (Li et al., 2022). However, no studies on the fertility of wild banana under temperature change have been reported. In our study, we found that pollens that were unable to germinate when stressed at low temperature were able to recover its germination capacity after 2 days of incubation at a constant of 28°C. In order to investigate the molecular mechanisms underlying the restoration of banana pollen fertility under the temperature change, this study constructed a regulatory network for the ability of wild banana pollens to restore germination by RNA-Seq of pollen that failed to germinate normally and was cultured at a constant temperature of 28°C for 2 days in Fujian, with the aim of providing a theoretical basis for further studies on the molecular mechanisms associated with wild banana pollens. In addition, some important candidate genes related to the ability of pollens to restore germination were identified in this study, which will provide theoretical support for further research on banana breeding.

## 2 Materials and methods

### 2.1 Plant materials

The wild banana used in this experiment came from the Fuzhou region. Mature male flower buds of wild banana were collected on a sunny day (23°C) in November. The outermost layer fresh pollens are first collected as untreated pollens. The buds, freed from the outer layer of pollens, are then inserted into water, where they continue to open once a day according to their original biological clock. They are then cultured at a constant temperature of 24°C, 28°C and 32°C for two days. After two days, the outermost pollen of the treated mature buds was collected and evenly distributed on the surface of the medium: [0.01% (w/v) boric acid + 12% (w/v) sucrose + 0.03% (w/v) calcium nitrate + 0.01% (w/v) potassium nitrate + 0.02% (w/v) magnesium sulphate + 0.8% (w/v) agar, pH 5.8-6.2], and observed after 24 hours cultured at a constant temperature of 28°C ( ± 1°C). The number of pollen tubes (length > 2 times the pollen diameter) was recorded. Three different fields of view were taken for each treatment group to calculate the pollen germination rate. Significant differences in pollen germination rates calculated using SPSS.

### 2.2 Total RNA isolation, mRNA library preparation and sequencing

The outer pollen of fresh buds collected as control material and the outer pollen of buds treated at 28°C for two days were used as treatment material for the comparative transcriptome sequencing analysis. The material was wrapped in foil and immediately fixed in liquid nitrogen after collection, and three biological replicates of each material were performed. The control groups were named CK1; CK2; CK3. The thermostatic treatment groups were named T1; T2; T3, and a total of six mRNA libraries were constructed. Total RNA of six groups were extracted from Fujian wild banana pollen by Jingjie Biological Company, and its concentration and purity were tested by agarose electrophoresis and Agilent 2100 Bioanalyzer, and the transcriptome was sequenced after passing the test.

Next generation sequencing (NGS), based on the Illumina sequencing platform, was used to sequence the pair-end (PE) of the constructed RNA library. The filtered reads were aligned to the reference genome using an enhanced version of HISAT2 software (http://ccb,jhu.edu/software/hisat2/index.shtml) prior to downstream analysis. The cleaned reads were aligned to the banana reference genome (https://banana-genome-hub.southgreen.fr/).

### 2.3 Analysis of differentially expressed genes

Expression was normalised using FPKM (Fagments Per Kilo bases per Million fragments), which is the number of fragments per kilobase length of a gene per million fragments. Differential expression gene (DEGs) analysis was performed using DESeq, with DEGs filtered by multiplicity of expression difference |log2FoldChange| > 1 and P-value < 0.05. GO enrichment analysis was performed using topGO, where the gene list and the number of genes per term were calculated using the differential genes annotated with GO terms, and then The P-value (significant enrichment is defined as a P-value < 0.05) was calculated by a hypergeometric distribution method to identify GO terms that were significantly enriched for the differential genes relative to the entire genomic background, thus identifying the main biological functions performed by DEGs. The results of the Kyoto Encyclopedia of Genes and Genomes (KEGG) pathway enrichment for DEGs were analysed through the KEGG databases. The FPKM of all DEGs analysed in this study show the average of the FPKM of the three biological replicates.

### 2.4 Transcription factors prediction and analysis

A 2000 bp sequence upstream of the promoter of 24 DEGs associated with pollen germination was extracted using TBtools, and the 24 DEGs associated with pollen germination were analysed for potential TFs using the online website JASPAR2020 (http://jaspar.genereg.net/), selecting *Arabidopsis thaliana* as the reference database. DEGs belonging to the MYB family were identified using the TBtools simple HMM search method. TFBS prediction for MYB using footprintDB2021 (http://floresta.eead.csic.es/footprintdb/index.php). Scanning of motifs up to 2000 bp upstream of the initiation codon for putative target genes was performed using FIMO (Find Individual Motif Occurences) in the Oxford University research programme of MEME suite (https://meme-suite./meme/index.html). Network graphs were constructed using Cytoscape software.

### 2.5 Determination of metabolite content

Cysteine content was determined using a test kit (HRK0733, Herui Bio, China); free fatty acid (FFA) content was determined using a test kit (HRK1311, Herui Bio, China); H_2_O_2_ content was determined using a test kit (HRK0521, Herui Bio, China); Fructose content was determined using a test kit (HRK1513, Herui Bio, China); D-Golactose content was determined using a kit (G0592W, Kemin, Suzhou, China); Sucrose content was determined using a kit (HRK1515, Herui Bio, China).

### 2.6 RT-qPCR analysis

First-strand cDNA was obtained by reverse transcription of total RNA using the Hifair^®^1st Strand cDNA Synthesis kit (Yeasen, Shanghai, China) kit, and the Roche LightCycler 480^®^ real-time PCR instrument (Roche Applied Science, Switzerland) was used for qPCR. A 20 μL reaction system with 50 cycles was designed with the reference (Ni et al., 2021); UBQ was used as an internal reference gene, and the 2^-ΔΔCT^ method (Lin and Lai, 2010) was used to calculate relative expression, and six differentially expressed pollen-associated genes were randomly selected for validation by qRT-PCR. All primers used in this study were listed in **Supplementary Table S1**.

## 3 Results

### 3.1 The effect of temperature change on pollen fertility in wild banana

**Figure 1:**
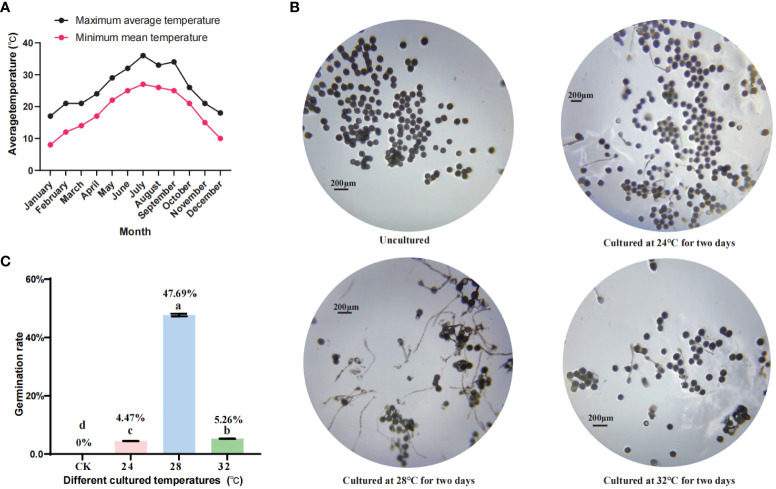
The effect of temperature change on pollen fertility in wild banana. **(A)** Average temperature from January to December of 2021 in Fuzhou, Fujian, China; **(B)** Germination of wild banana pollens at different temperatures (CK, 24°C, 28°C, and 32°C); **(C)** Germination rate of wild banana pollens at different temperatures (CK, 24°C, 28°C, and 32°C).

In this study, wild banana were grown in the Fuzhou area and the effect of temperature changes on pollen fertility was evaluated by statistically analysing historical weather changes in Fuzhou over the year (**Figure 1A**). First, we found that pollens failed to germinate from January to May and from October to December when the average minimum temperature was below 24°C (data not shown). As the pollen we collected in September did not germinate properly (data not shown), a statistical analysis of the temperatures from June to September showed that the minimum temperature in July and August was consistently around 27°C, with 27°C as the minimum temperature for more than 15 days, due to heavy rainfall during the typhoon season. 27°C was reached only one day in September, and the rest of the month was around 25°C-26°C, or even lower, so we believe that temperatures below 27°C may not be conducive to the germination of wild banana pollen. Considering that the optimum temperature for banana growth is 24-32°C, we set several temperature nodes (CK, 24°C, 28°C, and 32°C) to detect pollen viability in wild banana. The details are as follows: the outer pollen of fresh buds were collected as CK (23°C), and the outer pollen of buds treated at 24°C, 28°C, 32°C for two days were placed on basal *in vitro* germination medium to observed the germination of pollen tubes. We found that after a moderate increase in temperature, pollens cultured at 24°C, 28°C and 32°C for 2 days showed different degrees of germination capacity compared to freshly collected pollen, with pollens cultured at 28°C showing the highest germination capacity, followed by 32°C and finally 24°C (**Figures 1B, C**). It indicates that pollen stressed at low temperatures is able to recover its germination capacity at normal high ambient temperatures, but if the temperature is too high, it is not conducive to pollen germination. Therefore, we believe that 28°C can effectively promote pollen germination in wild banana compared to CK (23°C), 24°C and 32°C.

### 3.2 A comprehensive transcriptomic analysis of the pollen germination recovery process in wild banana

**Figure 2:**
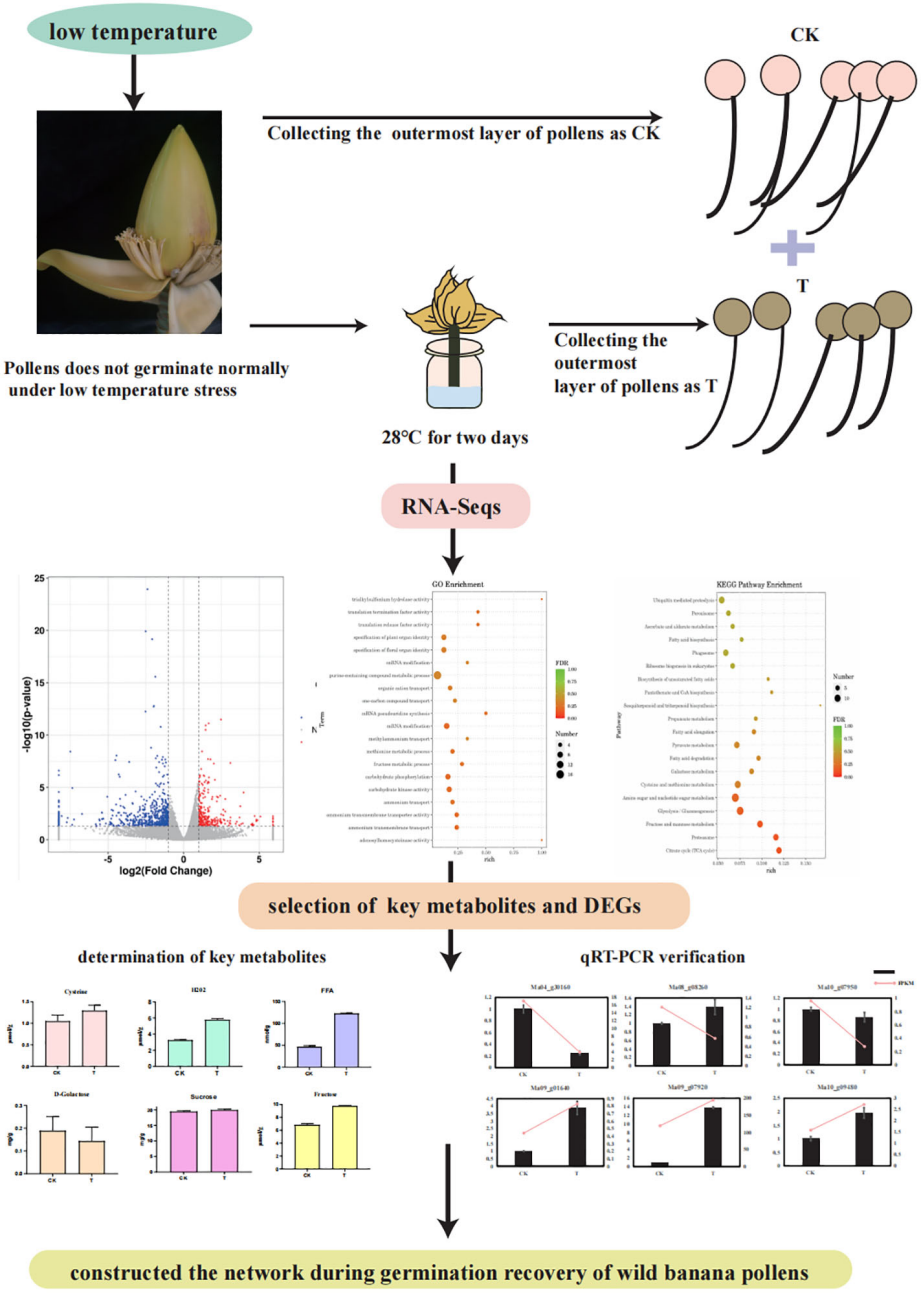
The transcriptome analysis process for this study.

**Figure 3:**
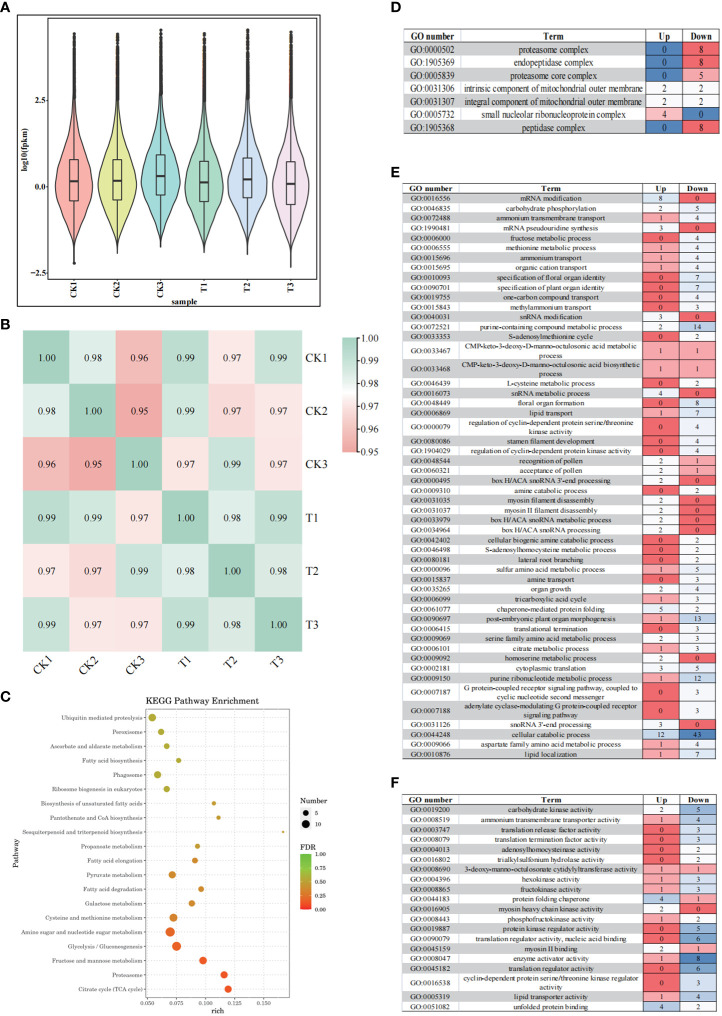
Complete transcriptome analysis of wild banana pollen under different temperature. **(A)** Violin plot representation of expression range from six libraries of two groups; **(B)** Pearson correlation between samples analysis; **(C)** Top 20 Kyoto Encyclopedia of Genes and Genomes (KEGG) enrichment pathway of CK vs T; **(D)** Gene Ontology (GO) analysis of DEGs in cellular components (CC) (p < 0.01); **(E)** Gene Ontology (GO) analysis of DEGs in biological processes (BP) (p < 0.01); **(F)** Gene Ontology (GO) analysis of DEGs in molecular functions (MF) (p < 0.01). The colour differences in the heatmap indicate differences in the number of DEGs in the different GO enrichment pathways.

The results showed that wild banana pollens had a higher germination capacity at 28°C compared to CK (23°C), 24°C and 32°C. To reveal the molecular mechanisms involved, the pollens of CK and the pollens treated at 28°C (named T) were collected to construct six mRNA libraries. Each sample contained three biological replicates. To ensure the quality of the sequencing data, we filtered the raw reads and performed sequence alignment analysis to calculate the number of genes, the research process for this study was as follows (**Figure 2**). A total of 42.13 GB of clean data was obtained from these 6 RNA-Seqs. The average clean data per sample was greater than 7.02 GB, with a Q20 percentage greater than 97.15% and a Q30 percentage greater than 93.23%, indicating that the accuracy and quality of the sequencing data was sufficient for further analysis (**Table 1**). Based on the FPKM values, the sample group expression violin plot was performed to show the expression distribution in the two groups of samples (**Figure 3A**). The dispersion of gene expression levels in the sample groups was moderate, with good overall abundance of gene expression between samples. The Pearson correlation coefficients were both equal to 1, indicating a strong correlation between the two sample groups of CK and T (**Figure 3B**).

**Table 1:** Summary of RNA-Seq data and sequence assembly.

sample	Raw read	Clean reads	Clean read (%)	Raw data (bp)	Clean data(%)	Q20 (%)	Q30 (%)	Unique Mapped reads	Mapped ratio
CK1	45679312	40807802	89.34	6851896800	89.33	97.56	93.83	25347974	94.20%
CK2	48906758	43731198	89.42	7336013700	89.41	97.61	94.04	26498317	90.40%
CK3	46577548	41684756	89.50	6986632200	89.49	97.27	93.33	22499324	93.00%
T1	47334246	42586360	89.97	7100136900	89.96	97.58	94.07	30409408	92.82%
T2	39246676	34723362	88.47	5887001400	88.47	97.15	93.23	23278880	92.46%
T3	53101636	47621616	89.68	7965245400	89.68	97.69	94.17	32716341	95.55%

### 3.3 Comprehensive profiling of DEGs analysis

To find out the DEGs between the CK and T, expression difference multiplicity | log2 (fold change) | > 1 and significance P-value < 0.05 were used as conditions for screening DEGs. A total of 921 DEGs were detected in the CK vs T, with 265 up-regulated expression and 656 down-regulated genes, the number of up-regulated genes being lower than the number of down-regulated genes in this study. To understand the function of DEGs, GO analysis was performed on these DEGs and the analysis revealed that these DEGs were mainly enriched in cellular components (CC) (**Figure 3D**), biological processes (BP) (**Figure 3E**) and molecular functions (MF) (**Figure 3F**). We analysed the significant enrichment pathways (p < 0.01) in each type of GO enrichment pathway (**Supplementary Table S2**). With p < 0.01, a total of 53 pathways were enriched in the biological processes (BP), including 14 metabolic pathways, including snoRNA, various amino acids and fructose, indicating the importance of metabolic processes. The biological processes (BP) contained seven processes related to the growth and development of floral organs (specification of floral organ identity, specification of plant organ identity, formation of floral organ, development of stamen filaments, pollen recognition, pollen acceptance, and organ growth). It also contains a variety of transport processes (transmembrane transport of ammonium, transport of ammonium, transport of organic cations, transport of a hydrocarbon compound, transport of methylammonium, transport of lipids, and transport of amines). A total of 7 pathways were enriched for cellular components (CC), mainly containing the protein complex pathway. A total of 20 pathways were enriched in molecular function (MF), mainly including activation of glycans (hexokinase activity, fructokinase activity, phosphofructokinase activity), and protein kinase activity (protein kinase regulator activity, cyclin-dependent protein serine/threonine kinase regulator activity). These results suggest that the activation, transport and metabolism of various substances (lipids, sugars, amino acids) play an important role in the restoration of pollen germination capacity in wild banana.

### 3.4 Analysis of KEGG metabolic enrichment pathways

A comprehensive analysis of the transcriptome found that the activation, transport and metabolism of various substances (lipids, sugars, amino acids) play an important role in the restoration of pollen germination capacity. We therefore focused the metabolic pathways of the three main groups of substances (lipids, sugars and amino acids) in the top 20 KEGG pathways (**Figure 3C**; **Supplementary Table S3**), in addition, we focused on the final metabolic pathway (TCA cycle) of lipids, sugars and amino acids, and on the most enriched sesquiterpenoid and triterpenoid biosynthetic pathway.

#### 3.4.1 DEGs involved in fatty acid metabolism in wild banana pollen

**Figure 4:**
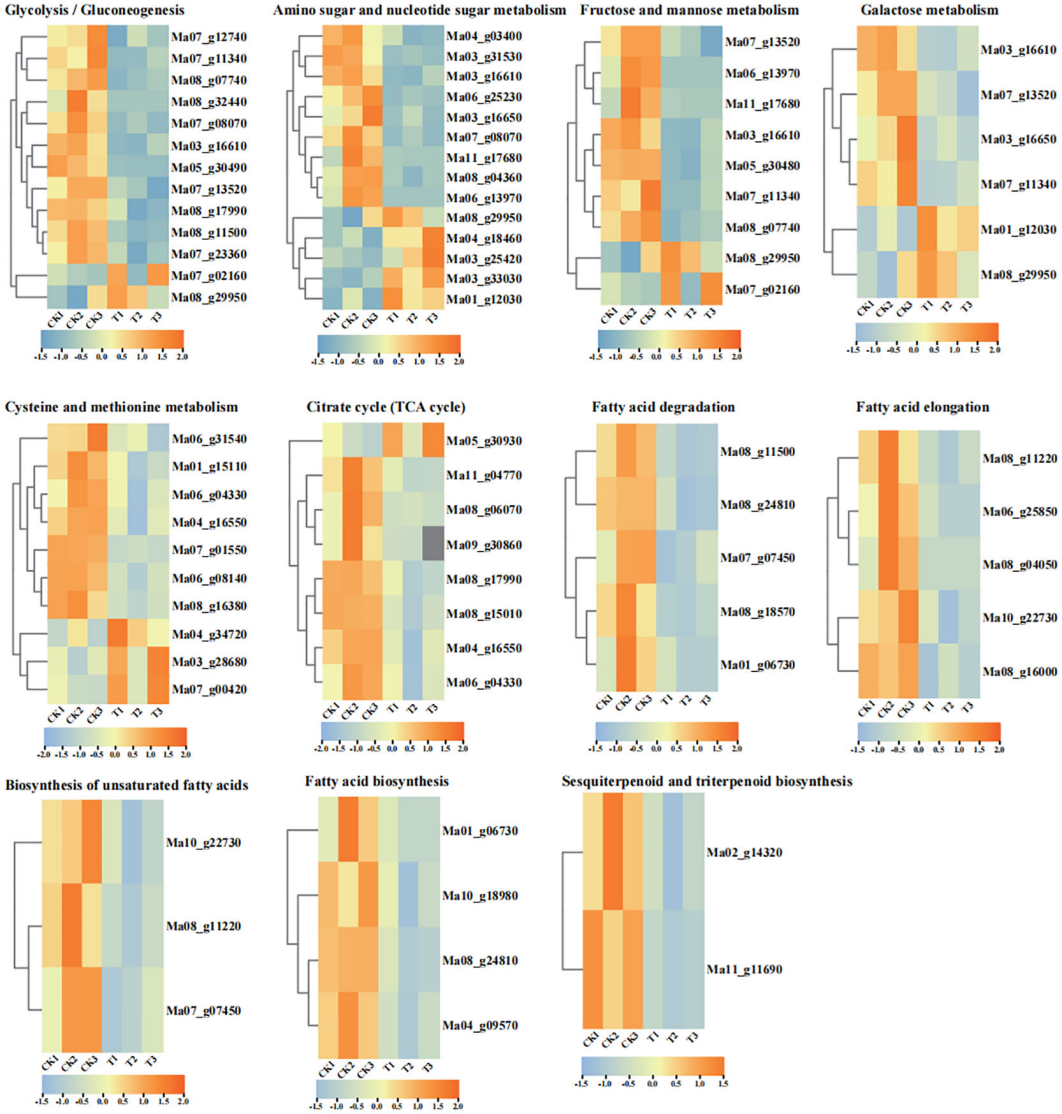
Heat map of the expression of DEGs in selected metabolic pathways of the top 20 KEGG pathways. FPKM values of all DEGs were normalized using Log2, and rows were clustered separately. Different colours indicate up-regulated expression or down-regulated expression of DEGs. All FPKM of DEGs analysed show the three biological replicates.

Fatty acid degradation, fatty acid elongation, biosynthesis of unsaturated fatty acids and fatty acid biosynthesis were enriched in CK vs T (**Supplementary Figures S1**–**S4**). *ALDH* [*aldehyde dehydrogenase (NAD+)*] (Ma08_g11500), *ACOX* (*acyl-CoA oxidase*) (Ma07_g07450), *CYP704B1* (Ma08_g18570), *LACS (long-chain acyl-CoA synthetase*) (Ma08_g24810, Ma01_g06730) were downregulated in the fatty acid degradation pathway. Very long-chain fatty acid (3R)-3-hydroxyacyl-CoA dehydratase (Ma10_g22730), *ACOT1_2_4* (*acyl-coenzyme A thioesterase 1/2/4*) (Ma08_g11220), *KCS* (*3-ketoacyl-CoA synthase*) (Ma06_g25850, Ma08_g04050), and *MECR* (*mitochondrial enoyl-[acyl-carrier protein] reductase*) (Ma08_g16000) are downregulated in the fatty acid elongation pathway. *LACS* (*long-chain acyl-CoA synthetase*) (Ma08_g24810, Ma01_g06730), *ACACA (acetyl-CoA carboxylase/biotin carboxylase 1*) (Ma10_g18980), and *ACC* (*acetyl-CoA carboxylase*) (Ma04_g18980) were downregulated in the fatty acid biosynthetic pathway, *very long chain (3R)-3-hydroxyacyl-CoA dehydratase* (Ma10_g22730), *ACOT1_2_4* (Ma08_g11220), and *ACOX* (Ma07_g1130) were downregulated in the biosynthesis of unsaturated fatty acids pathway (**Figure 4**, **Supplementary Table S4**). In general, all DEGs showed downregulated expression in fatty acid-related pathways, which may be associated with fatty acid accumulation. Among them, *KCS* (Ma06_g25850), *MECR* (Ma08_g16000) and *very long chain (3R)-3-hydroxyacyl-CoA dehydratase* (Ma10_g22730) were significantly downregulated in the Fatty acid elongation. We found that *MECR* can affect the synthesis of various CoAs (Hexanoyl-CoA, Octanoyl-CoA, Decanoyl-CoA, Dodecanoyl-CoA, Tetradecanoyl-CoA, and Hexadecanoyl-CoA), which in turn affects downstream fatty acid synthesis. In addition, we found that the synthesis of long-chain fatty acids could influence the downstream biosynthesis of cutin, suberin and wax, in which *CYP704B1* plays a role, and its slight down-regulation at 28°C may be related to changes in pollen wall synthesis during pollen germination.

#### 3.4.2 DEGs involved in carbohydrate/sugar metabolism in wild banana pollen

In total, four pathways of sugar metabolism were enriched: fructose and mannose metabolism, glycolysis/gluconeogenesis, amino sugar and nucleotide sugar metabolism and galactose metabolism (**Supplementary Figures S5**–**S8**). The glycolysis/gluconeogenesis pathway was enriched by 2 upregulated and 11 downregulated DEGs; fructose and mannose metabolism was enriched by 2 upregulated and 7 downregulated DEGs, amino sugar and nucleotide sugar metabolism were enriched for a total of 5 upregulated and 9 downregulated DEGs, and galactose metabolism was enriched for a total of 2 up-regulated and 4 down-regulated DEGs (**Figure 4**). *PFP* is capable of being present in glycolysis and gluconeogenesis and can influence the allocation of carbohydrates to sugars and organic acids (Basson et al., 2011) and is at the intersection of the metabolic pathways of glycolysis/gluconeogenesis and fructose and mannose metabolism. *HXK* (hexokinase) is involved in hexose phosphorylation and plays an important role in fructose accumulation (Zhang et al., 2014), and is the link of amino and nucleotide sugar metabolism, glycolysis and gluconeogenesis, galactose metabolism and fructose and mannose metabolism, and is responsible for the synthesis of α-D-Glucose-6P and β-D-Glucose-6P. We found that some DEGs encoding hexokinase are upregulated and others are downregulated, suggesting a complex mechanism for *HXK* in sugar metabolism. Next, we focused on several DEGs in the metabolism of amino sugars and nucleotides associated with glycogen synthesis in the cell wall, including *UDP-glucose 4,6-dehydratase* (Ma03_g33030), *UDP-glucuronate 4-epimerase* (Ma04_g18460), and *UDP-glucose 4-epimerase* (Ma01_g12030), all of which are upregulated in the amino sugar and nucleotide sugar metabolism pathways. Among them, *UDP-glucose 4-epimerase* was also enriched in galactose metabolism, and it may play a role in linking galactose metabolism with amino and nucleotide sugar metabolism pathways, suggesting that both galactose metabolism and amino and nucleotide sugar metabolism play important roles in maintaining cell wall integrity. In addition, we have identified two DEGs whose expression is significantly down-regulated in the glycolysis/gluconeogenesis pathway: *2,3-bisphosphoglycerate-dependent phosphoglycerate mutase* (*gpmB*) (Ma07_g12740) and *2,3-bisphosphoglycerate-independent phosphoglycerate mutase* (*gpmI*) (Ma07_g23360) are involved in the glycolytic and gluconeogenic pathways and are jointly responsible for the conversion between 3-P glycerate and 2-P glycerate.

#### 3.4.3 DEGs involved in cysteine and methionine metabolism in wild banana pollen

There are three upregulated DEGs (Ma04_g34720, Ma03_g28680, and Ma07_g00420) and seven downregulated DEGs (Ma06_g04330, Ma01_g15110, Ma06_g31540, Ma06_g16550, Ma08_g16380, and Ma07_g01550) in the cysteine and methionine metabolism pathway (**Figure 4**, **Supplementary Figure S9**). ThrA (Ma04_g34720) (bifunctional aspartokinase/homoserine dehydrogenase 1), a bifunctional enzyme that catalyses the first step of lysine and homoserine biosynthesis and indirectly methionine and threonine biosynthesis, as well as the final step in homoserine biosynthesis. *DNMT1* (*DNA (cytosine-5)-methyltransferase 1*) whose main role *in vivo* is to maintain the methylation level of the organism. Its homologous gene in plants is *MET1* (*methyltransferase 1*) (Ma03_g28680 and Ma07_g00420). It is hypothesised that it may play a role in maintaining methylation levels during pollen germination recovery. Regarding genes whose expression is down-regulated, we found that S-adenosyl-l-homocysteine Hydrolase (*SAHHs*) (Ma08_g16380 and Ma06_g08140) whose expression is significantly down-regulated at 28°C. S-adenosyl-L-homocysteine hydrolase (SAHH) is a key enzyme in the regulation of intracellular methylation reactions. Overall, *DNMT1* and *SAHHs*, enriched in the cysteine and methionine metabolism pathway, may play an important role in maintaining methylation levels during pollen germination.

#### 3.4.4 DEGs involved in citrate cycle (TCA cycle) in wild banana pollen

The TCA cycle is the final metabolic pathway for the three major nutrients (lipids, sugars and amino acids). In total, we detected one up-regulated and seven down-regulated expression genes in the TCA cycle (**Supplementary Figure S10**). *OGDH* (*E1 component of 2-oxoglutarate dehydrogenase*) (Ma05_g30930) was up-regulated and the *ACO* (*aconitate hydratase*) (Ma09_g30860), *ACLY* [(*ATP citrate (pro-S)-lyase*)] (Ma11_g04770, Ma08_g06070), *DLAT* (*dihydrolipoamide acetyltransferase*) (Ma08_g17990), *LSC1* (*succinyl-CoA synthetase alpha subunit*) (Ma08_g15010), and *MDH* (*malate dehydrogenase*) (Ma06_g04330, Ma04_g16550) were down-regulated (**Figure 4**). *OGDH* is one of the key enzymes involved in the fourth step of the TCA, catalysing the oxidative decarboxylation of α-ketoglutarate to produce succinyl coenzyme A. Therefore *OGDH* is upregulated in the TCA cycle and may promote the subsequent synthesis of succinyl coenzyme A. In addition, we found 2 differentially expressed enzymes-ACLYs. We are concerned that simultaneous down-regulation of *ACLY* and *MDH* may affect oxaloacetate production and thus the downstream pathway of glycolysis/gluconeogenesis.

#### 3.4.5 DEGs involved in sesquiterpenoid and triterpenoid biosynthesis in wild banana pollen

Furthermore, we focused on the sesquiterpenoid and triterpenoid biosynthetic pathway, and found that acetyl-CoA ultimately forms farnesyl pyrophosphate through a series of reactions, and once farnesyl pyrophosphate has been synthesised, the sterol pathway and the non-sterol pathway are initiated (Ha and Lee, 2020). We found that the expression of *FDFT1* (farnesyl-diphosphate farnesyltransferase) (Ma02_g14320, Ma11_g11690) was down-regulated at 28°C (**Figure 4**), and we suggest that the down-regulated expression of *FDFT1* may affect Squalene synthesis and further influence the steroid synthesis process.

### 3.5 Hormonal signaling during pollen germination in wild banana

**Figure 5:**
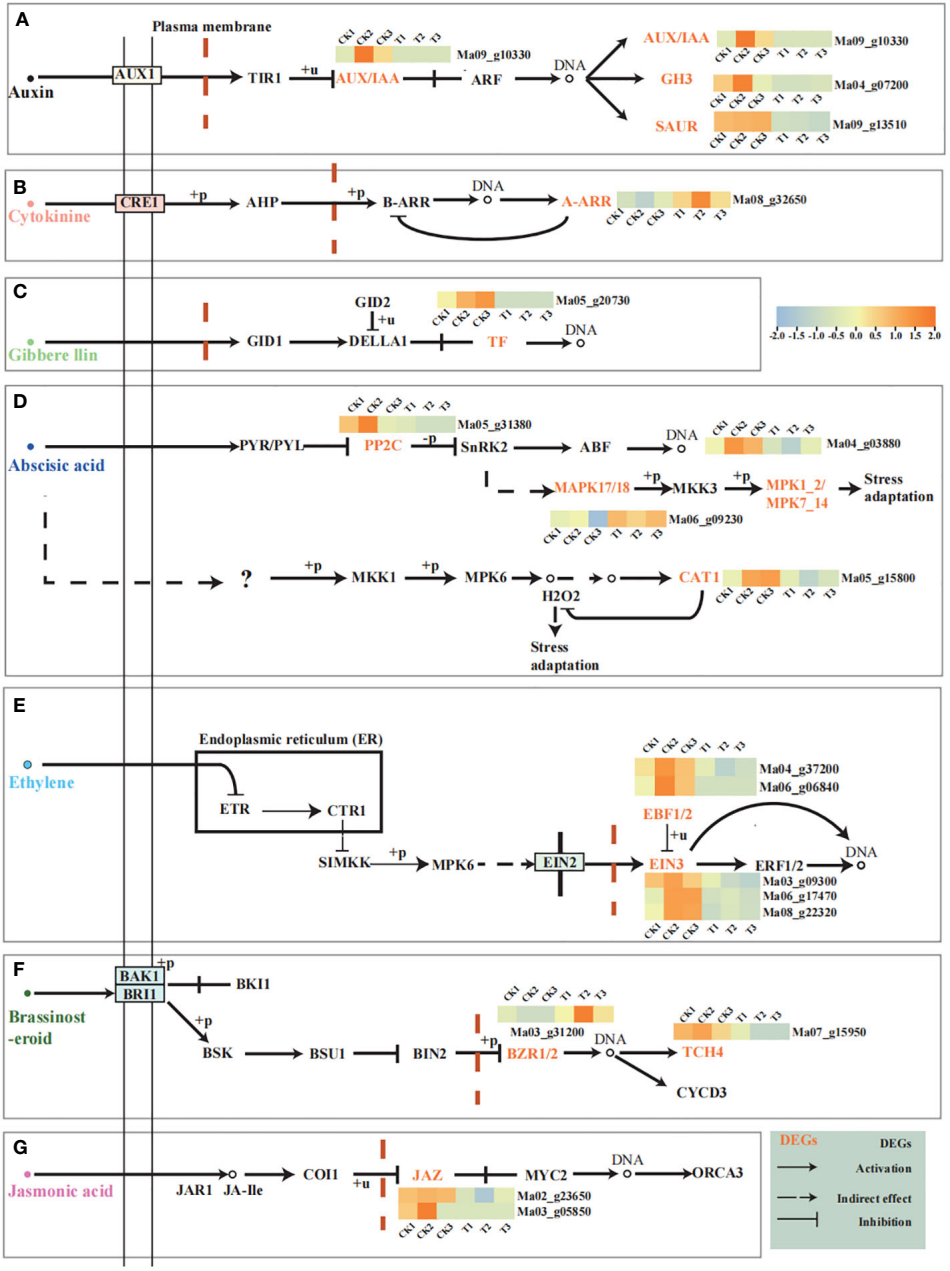
Hormonal signalling during pollen germination. **(A)** Auxin signalling during pollen germination; **(B)** Cytokinine signalling during pollen germination; **(C)** Gibberellin signalling during pollen germination; **(D)** Abscisic acid signalling during pollen germination; **(E)** Ethylene signalling during pollen germination; **(F)** Brassinosteroid signalling during pollen germination; **(G)** Jasmonic acid signalling during pollen germination; The light yellow boxes indicate DEGs. Heat map indicates the expression of DEGs. The hormonal signalling network is designed with reference to the KEGG pathway. All FPKM of DEGs analyzed show the average of the FPKM of the three biological replicates.

To further understand the role of hormones in the process of pollen germination (**Figure 5**). Based our analysis on the phytohormone transduction pathway. We found that wild banana was able to respond to Auxin, Cytokinine, Gibbere llin, Abscisic acid, Ethylene, Brassinosteroid and Jasmonic acid hormone signaling pathways during pollen germination. We found that *A-ARR* (Ma08_g32650) and *BZR1/2* (Ma03_g31200) were up-regulated in the Cytokinine and Brassinosteroid pathways, respectively, and the rest of the genes were down-regulated in each hormone pathway. Auxin pathways showed up-regulation of *AUX/IAA* (Ma09_g10330), *GH3* (Ma04_g07200) and *SUAR* (Ma09_g13510) were down-regulated, with *SUAR* showing significant down-regulation; *PIF3* (Ma05_g20730) was down-regulated in the Gibberellin pathway; *PP2C* (Ma05_g31380) was down-regulated in the Abscisic acid pathway; *EBF1_2* (Ma04 g37200, Ma06_g06840), *EIN3* (Ma06_g17470, Ma08_g22320, and Ma03_g09300) showed down-regulation in the Ethylene pathway; *TCH4* (Ma07_g15950) and *JAZ* (Ma02_g23650 and Ma03_g05850) were down-regulated in the Brassinosteroid and Jasmonic acid hormone signaling pathways, respectively. Overall, the hormone-related genes in pollen germination of wild banana showed an overall trend of down-regulated expression.

MAPK signalling is also important for pollen germination. *MAPKKK17_18* is up-regulated in the downstream pathway of ABA signalling, while *MPK1_2* and *CAT1* are down-regulated. We suggest that the upregulation of *MAPKKK17_18* may promote phosphorylation of *MKK3* and thus reduce expression of *MPK1_2*, while the reduction of *CAT1* may promote H2O2 synthesis (**Figure 5D**).

### 3.6 Determination of key metabolites and qRT-PCR analysis of DEGs

In this study, we found that wild banana pollens involves multiple metabolic pathways during germination. We therefore measured the key metabolites of certain pathways. Sugar metabolism is an important pathway during pollen germination, and we found that, compared with CK, sucrose and fructose content increased and galactose content decreased in pollen treated at 28°C for two days. The accumulation and uneven distribution of H_2_O_2_ is necessary for pollen germination in some plants (Maksimov, 2018), and we found an increase in H_2_O_2_ content under a 28°C treatment, which could be linked to temperature change-induced pollen germination in wild banana. In addition, the cysteine content also increased, which may be due to the enrichment of cysteine in pollen coat during germination (Doughty et al., 2000). The FFA content in treated pollen also increased compared with CK, which may be linked to the energy balance of the pollen germination process.

**Figure 6:**
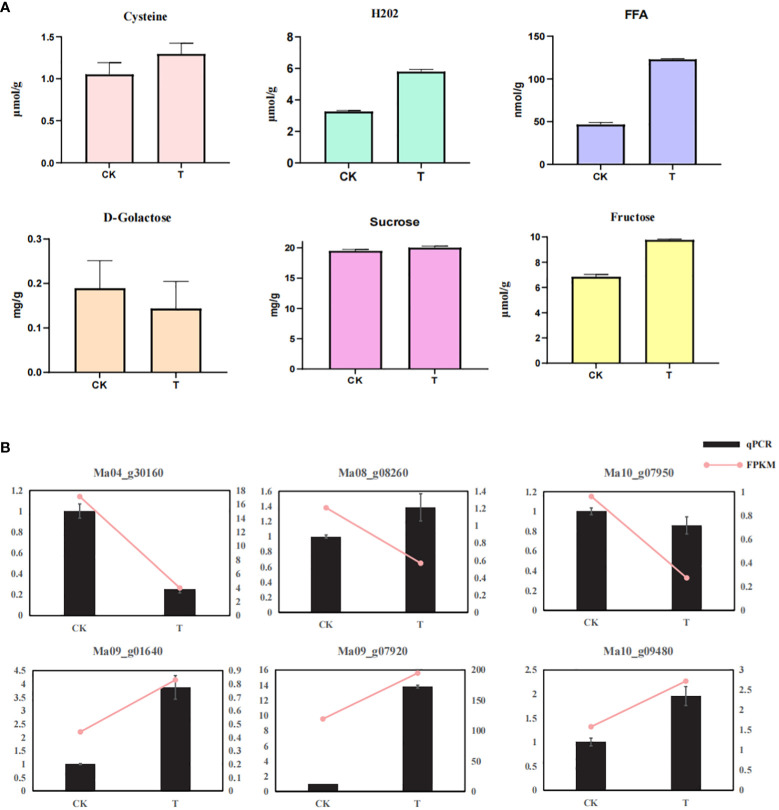
Determination of key metabolites and qRT-PCR analysis of DEGs. **(A)** Determination of key metabolites of CK and T; **(B)** qRT-PCR validation of six randomly selected gene.

Meanwhile, to validate the reliability of transcriptome expression, six genes were randomly selected from the DEGs for qRT-PCR analysis, including Ma10_g09480, Ma09_g07920, Ma04_g30160, Ma09_g01640, Ma08_g02860, and Ma10_g07950. qRT-PCR results showed that the expression patterns of these six genes were basically consistent with the transcriptome data (**Figure 6**), demonstrating that the transcriptome is highly reliable.

### 3.7 Identification and analysis of genes involved in the recovery of pollen germination

#### 3.7.1 Identification and expression analysis of DEGs related to the recovery of pollen germination in the GO pathway

The process of recovery of pollen germination necessarily involves the activation of a series of genes associated with pollen development. We searched for genes related to this progress based on GO enrichment files using the keyword ‘pollen’ and found a total of 16 related pathways, four of which were significantly enriched (p<0.05), resulting in 24 DEGs (**Supplementary Table S5**). In total, five DEGs were upregulated in the pollen germination-related pathway: *the mitochondrial-like 3-deoxy-mano-octulosonate cytidylyltransferase* (Ma04_g08050), *the probable carboxylesterase 18* (Ma09_g07920), and *PREDICTED: Kinesin-like protein KIN12B* (Ma09_g01640), *X1 isoform of small nucleolar RNA-like protein 2-like U3* (Ma08_g02860), and *Small nucleolar RNA interaction protein 2-like U3* (Ma10_g09480). These genes are involved in biological processes such as ribosome biosynthesis, nucleotide sugar biosynthesis, catalytic ester and amide compounds, gibberellin biosynthesis and cell growth. We analysed the expression of these genes and found a clear upregulation trend for the Ma09_g07920, suggesting that the pathway involved in the probable carboxylesterase 18 plays an important role in pollen germination. In total, 19 down-regulated genes were enriched in 16 pathways: Ma04_g30160 was enriched in six pathways: pollen tube, pollen tube growth, pollen tube development, pollen sperm cell differentiation, pollen development, and regulation of pollen tube growth, suggesting that it may play an important role in pollen germination and pollen tube growth. *CYP704B1* (Ma08_g18570) was enriched in three pathways, pollen exine formation, pollen wall assembly and sporopollenin biosynthesis process, and *CYP704B1* is essential for sporopollenin synthesis in *Arabidopsis* pollen. It is a long-chain fatty acid-ω-hydroxylase essential for sporopollenin synthesis in *Arabidopsis* pollen, and plays an important role in pollen wall formation (Dobritsa et al., 2009). *The probable beta-1,4-xylosyltransferase IRX9H* (Ma11_g21240) and *the probable galacturonosyltransferase 13 isoform X1* (Ma05_g01550) were found enriched in several pathways. *HMG B9* (*high mobility group B protein 9-like*, Ma06_g22410), which was found to be enriched in pollen tube, pollen tube growth, pollen tube growth, pollen tube development, and pollen germinatio. It should be noted that Ma06_g28660 and Ma04_g08050 are both 3-deoxy-mano-octulosonate cytidylyltransferases, the former being downregulated in pollen tube growth, pollen tube development and pollen development, while the latter is upregulated in these pathways. We assume that both are responsible for opposite functions in these pathways (**Supplementary Table S5**).

#### 3.7.2 Regulatory network of MaMYB involved in pollen germination process

**Figure 7:**
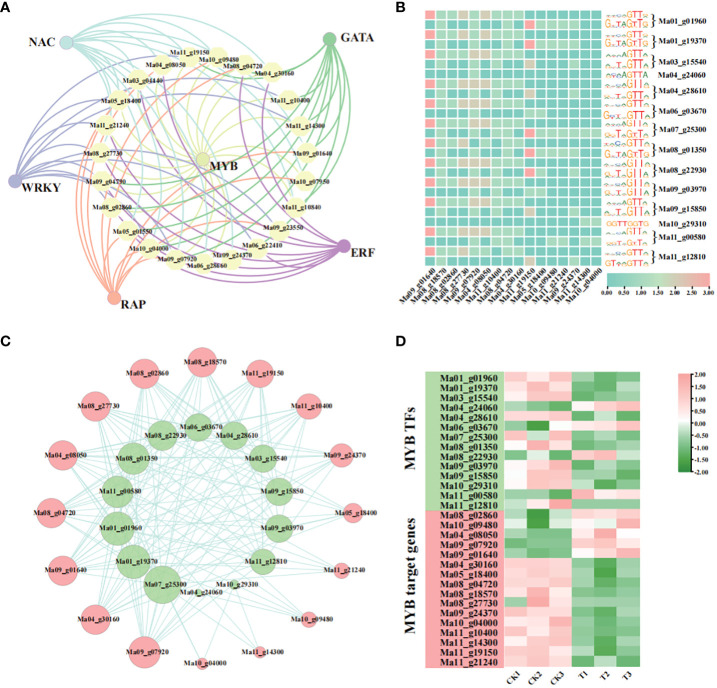
Regulatory network analysis of GO-enriched pollen germination associated DEGs. **(A)** Regulatory network of TFs of GO-enriched pollen germination-associated DEGs; **(B)** MYBs targeting pollen germination-associated genes and associated potential TFBS; **(C)** Regulatory network of MYBs and pollen germination-associated genes (where green represents MYBs and pink represents pollen germination-associated genes); **(D)** Expression analysis of MYBs and pollen germination-associated genes.

According to previous reports, TFs are also involved in the pollen development process. We performed a predictive analysis of TFs likely to be subject to possible regulation of genes associated with the recovery of pollen germination in the GO enrichment pathway described above. The results showed that 24 DEGs were targeted and regulated by a total of 72 TFs. Among them, MYBs were the main TFs, they mainly regulate genes whose expression is down-regulated (**Figure 7A**, **Supplementary Table S6**). We suggest that MYB may play an important role in the pollen germination process. Therefore, we combined DEGs to identify MYBs that regulate DEGs which related to the recovery of pollen germination. We performed a TFBS enrichment analysis of 14 differentially expressed MYBs by footprintDB2021 (http://floresta.eead.csic.es/footprintdb/index.php) (**Supplementary Table S7**). The results showed that Ma_01_g01960 and Ma_01_g19370 were enriched in the same DNA binding sites, including rwwcaGTTr and GtTAGTTG. Many members were enriched in the rwwmaGTTr DNA binding site. For example, Ma04_g28610, Ma06_g03670, Ma08_g22930, Ma09_g03970, and Ma11_g00580. In contrast, rwwmAGTTA was only enriched in Ma04_g24060 and Ma11_g00580. GGTTGGTG was only enriched in Ma10_g29310. In addition, we analysed the promoter sequences of 24 pollen germination-associated DEGs (2 000 bp upstream of the ATG) using FIMO (Find Individual Motif Occurences) in the MEME suite and a total of 16 DEGs were detected as being targeted and regulated by MYB TFs (**Figure 7B**, **Supplementary Table S8**). To this end, we constructed a MYB transcriptional regulatory network with 16 DEGs associated with pollen germination (**Figure 7C**), and found that the entire expression network involved genes enriched in multiple GO pathways, including pollen exine formation, pollen wall assembly, sporopollenin biosynthetic process, pollen development, and other pathways. We then analysed the expression levels of MYBs and their target genes, and found that a gene can be regulated by several MYBs, with some MYBs coinciding with the expression of the gene, e.g. Ma01_g19370 vs. Ma09_g07920, and others in contrast, e.g. Ma04_g24060 vs. Ma09_g07920. This implies a complex regulatory network of pollen germination processes involving TFs (**Figure 7D**, **Supplementary Table S8**).

## 4 Discussion

### 4.1 Various metabolic pathways regulate the germination process of wild banana pollen

**Sugar metabolism:**Sugars are mainly generated from the resource organs and transported into the sink organs functioning as both the reserved energy and essential cell compounds to promote callose wall and primexine formation, intine development, pollen maturation and starch accumulation, and pollen germination and tube growth (Liu et al., 2021). Studies have shown that different types of sugar have different effects on pollen germination. Glycolysis was shown to regulate growth polarity in *Arabidopsis* pollen tubes via impingement of Rho GTPase-dependent signalling (Wei et al., 2018). Fructose inhibited the germination of pear pollen *in vitro*, and this inhibition was dose-dependent (Okusaka and Hiratsuka, 2009). Hexoses such as glucose and mannose also inhibited pollen germination (Hirsche et al., 2017), and similarly, galactose metabolism inhibited pollen germination (Wang et al., 2022). During germination of wild banana pollen, the content of endogenous fructose as well as sucrose increases and the content of galactose decreases (**Figure 6**). In addition, we focused on a number of DEGs in these metabolic pathways, with *HXK* upregulated in the Glycolysis/Gluconeogenesis pathway, which may promote phosphorylation of hexoses, thus allowing phytoplankton to enter glycolysis and provide energy for growth. In addition, a number of DEGs associated with cell wall glycogen synthesis are upregulated in various metabolic pathways, including *UDP-glucose 4,6-dehydratase* (Ma03_g33030), *UDP-glucuronate 4-epimerase* (Ma04_g18460), and *UDP-glucose 4-epimerase* (Ma01_g12030), the upregulation of these DEGs may provide a material basis for pollen wall formation.

**Fatty acid metabolism: **The pollen wall is usually divided into an exine and an intine played a vital role in growth of pollen. Fatty acids and their derivatives are an important component of anther cuticle and pollen wall formation. This suggests that lipid metabolism plays a key role in the anther cuticle and pollen wall. The DEGs in the lipid metabolism pathways of top 20 KEGG pathways all showed a down-regulated expression pattern. This may be due to the fact that low temperature stress may accelerate the metabolism of lipids. This phenomenon may help pollen maintain internal heat and resist cold stress. Once when the temperature returns to normal levels, metabolism-related gene expression returns to normal levels and pollen regains its ability to germinate. In addition, we identified a number of lipid metabolism-associated DEGs, such as *LACs* (Ma08_g24810, Ma01_g06730). In previous studies, *LACS* can provide CoA-activated v*ery long chain fatty acids* (VLCFA-CoAs) for wax biosynthesis. *Arabidopsis lacs1 lacs4* double knockout mutant plants exhibit conditional sterility and a significant reduction in pollen sebum (Jessen et al., 2011). We therefore suggest that differential expression of *LACs* may contribute to cuticle formation during the resumption of pollen germination.

**TCA cycle:** The TCA cycle plays a central role in the overall regulatory metabolic network, with changes in metabolic pathways between all three major classes interacting with each other. *ODGH*, which is only one upregulated in the TCA cycle may further facilitate the production of succinyl CoA. The thioester bond of succinyl CoA is hydrolysed, releasing free energy for the synthesis of GTP, which in bacteria and higher organisms produces ATP directly, and in mammals, first into GTP and then into ATP. We therefore suggest that the rise of *ODGH* may have facilitated vegetative germination. Interestingly, human *OGDH* was found to be a novel calcium-binding site (Armstrong et al., 2014), and this calcium-binding site is essential for pollen development in plants, where Ca-binding proteins are generally able to be expressed in the pistil and anthers of flowers (Furuyama and Dzelzkalns, 1999). In addition, ogdh in *Arabidopsis* that reduce respiration rates, affect photosynthesis and ultimately carbon and nitrogen metabolism (Condori-Apfata et al., 2019). Once carbon and nitrogen metabolism is affected, this can lead to a decrease in carbohydrate synthesis. Consequently, up-regulation of *OGDH* during pollen germination can favour carbohydrate metabolism, which in turn favours the level of accumulation of sugar sources and provides the nutrients required for pollen germination.

### 4.2 DNA methylation and DNA repair during the recovery of pollen germination process

**Figure 8:**
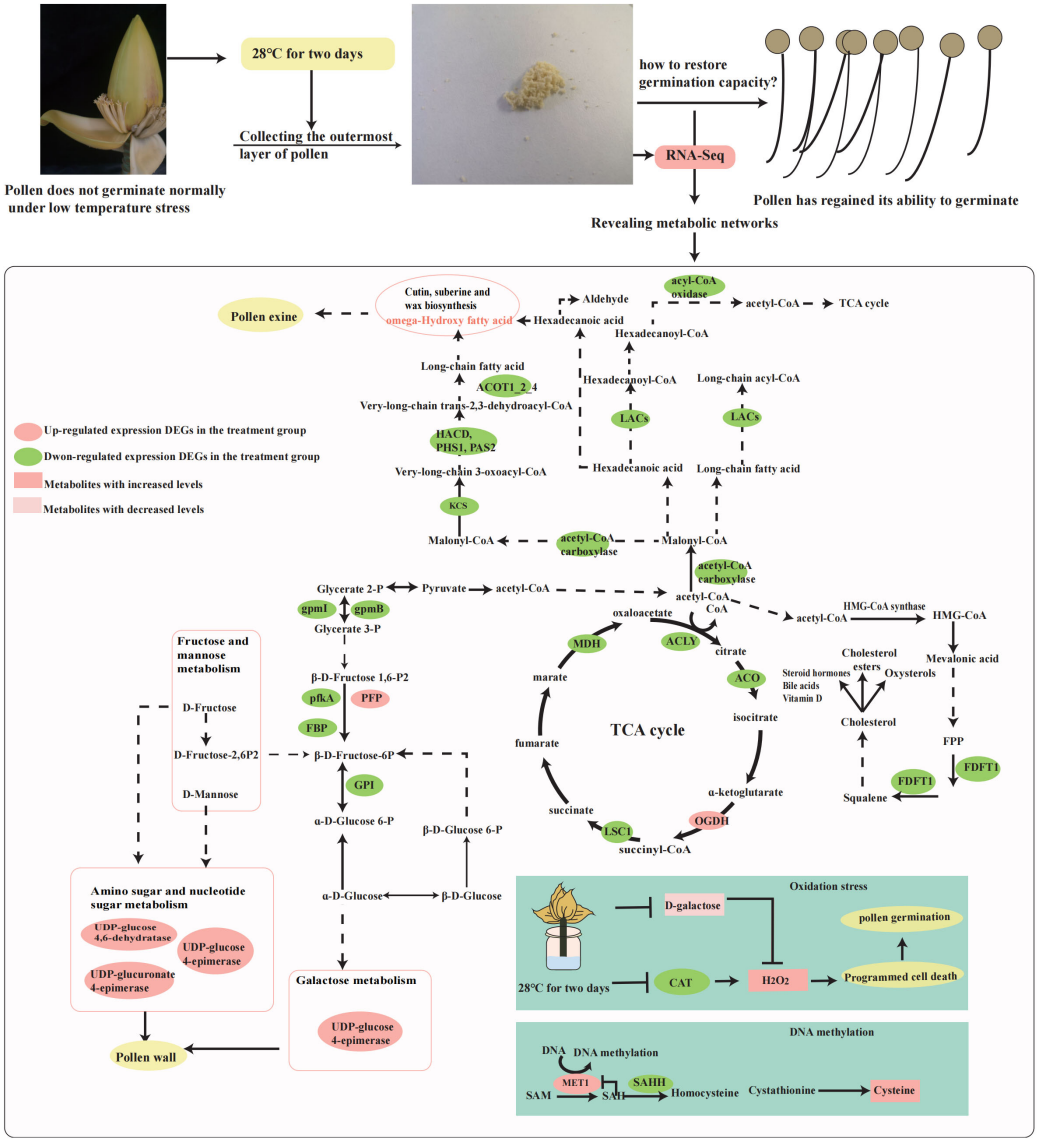
The main metabolic network during germination recovery of wild banana pollen. The metabolic network is designed with reference to the KEGG pathway. Where solid arrows indicate direct synthesis and dashed lines indicate indirect synthesis.

A growing number of studies have shown that DNA methylation is essential for plant growth and development. With the development of molecular biology, the relationship between DNA methylation and fertility has also gradually been revealed. Cotton pollen dieback may be associated with DNA methylation abnormalities (Kong et al., 2020). DNA methylation levels in fertility-restored hybrids were higher than those in infertility, and they suggested that DNA methylation may be involved in regulating the expression of CMS-C fertility restoration in maize (Chen et al., 2016). In Arabidopsis, DME (DEMETER) and ROS1 (REPRESSOR OF SILENCING 1) act in a semi-redundant manner in pollen feeder cells to DNA demethylate and ensure good pollen tube conductivity (Khouider et al., 2021). This suggests that the dynamics of DNA methylation are critical for the maintenance of fertility. For flowering plants, the DNA base excision repair process can facilitate DNA demethylation (Gehring et al., 2009). APEs are involved in DNA damage repair during pollen meiosis, and double mutations in APE1L and APE2 can lead to increased chromosome fragmentation during pollen meiosis and ultimately pollen abortion, suggesting that DNA repair is important for pollen fertility (Li et al., 2023). These results suggest that DNA methylation and repair support the maintenance of fertility. In this study, we identified two members in the cysteine and methionine metabolism pathway whose expression is significantly down-regulated: the SAHHs (Ma08_g16380 and Ma06_g08140) (**Figure 4**). SAHHs play an role in trans-methylation processes. Plants require a number of trans-methylation processes during growth. The trans-methylation process requires methyltransferases to be carried out, and the maintenance of methyltransferase activity requires S-adenosyl-L-methionine (SAM) as a methyl donor. However, S-adenosyl-L-homocysteine (SAH), a by-product of the methylation process, inhibits methyltransferase activity. Therefore, to ensure correct methylation, SAHH is able to rapidly convert SAH to L-homocysteine (HCY) and adenosine (ADO (**Figure 8**) (Alegre et al., 2020). We therefore suggest that the significant down-regulation of SAHHs at 28°C may affect methylation levels during pollen germination. However, we found that MET1 (Ma03_g28680 and Ma07_g00420), which is a methyltransferase, exhibited up-regulated expression in this pathway, which may be due to the other factors. MET1 maintains CG methylation, which is essential for TE (transposon) silencing (Wang and Baulcombe, 2020). In pollen, this is where transposon control becomes really critical because genomic changes are passed on to the progeny. So, during pollen germination, up-regulation of MET1 expression can promote CG methylation to better control transposons. Interestingly, in mammals, *DNMT1(MET1 homolog)* and *AHCY (SAHHs homolog)*are involved in the DNA damage and repair process, with a decrease in *AHCY* leading to an increase in DNA damage (Belužić et al., 2018), while *DNMT1* is able to be recruited to the DNA repair site in response to DNA damage (Mortusewicz et al., 2005). We speculate whether MET1 and SAHHs are also involved in the process of DNA repair in wild banana pollen germination to ensure normal pollen germination.

### 4.3 Oxidation stress during the recovery of pollen germination process

ROS play an important role in the formation of viable pollen (Xie et al., 2022). Inhibition of ROS accumulation also severely disrupted the actin cytoskeleton in pollen tubes (Pasqualini et al., 2015). The programmed cell death (PCD) of chorioallantoic cells during microspore development in Cupressus arizonica involves the role of ROS (Xie et al., 2022). ROS levels in *Arabidopsis* and rice anthers peak at the time of chorion denaturation and pollen maturation (Hu et al., 2011; Xie et al., 2014; Yi et al., 2016). H_2_O_2_ levels were up-regulated and CAT expression was decreased in wild banana pollen by the treatment (**Figures 5**, **6**). H_2_O_2_ is considered to be a major component of ROS, CAT can catalyze the decomposition of hydrogen H_2_O_2_. So we suggest that an increase in H_2_O_2_ content and decrease of CAT expression will lead to an increase in the level of ROS and then occurs the oxidation stress. It was found that mild heat stress could also induce ROS production and activate programmed cell death in the chorioallantoic layer in time to promote pollen germination (Rieu et al., 2017). In this study, wild banana pollens were incubated at a constant temperature of 28°C for 2 days and subjected to mild heat stress relative to CK, resulting in an increase in ROS content to promote pollen germination. However, only an adequate amount of ROS will further promote pollen germination. The reduced ability of wild banana pollen to germinate under 32°C treatment (**Figures 1B, C**) may be due to the excessive accumulation of ROS, which inhibits pollen tube elongation (Muhlemann et al., 2018). Furthermore, we found that D-galactose content was reduced in wild banana pollen at 28°C. It has been reported that D-galactose can increase H_2_O_2_ levels and therefore trigger oxidative damage (Azman and Zakaria, 2019). The reduced D-galactose content was reduced under 28°C treatment, which could control H_2_O_2_ to a stable level by suppressing the H_2_O_2_ level that was elevated after being subjected to mild heat stress, then maintain the oxidative stress homeostasis during pollen germination.

### 4.4 Pollen wall remodelling may be important for the resumption of pollen germination in wild banana

Pollen grains are enclosed in a multi-layered pollen wall. The pollen wall is important for the function of the pollen and studies have shown that it helps the pollen to resist a number of external stresses such as heat, ultraviolet radiation, microbial attack, water loss and so on (Ariizumi and Toriyama, 2011). The pollen wall is generally divided into the inner intine and the outer exine. The outer exine is divided into an outer and an inner layer. The outer exine contains mainly sporopollenin, which is strong and resistant to acids and biodegradation. Lipids are the main components of the pollen wall and the synthesis of lipid precursors such as sporopollenin, keratin and wax is essential for pollen wall development (Yuan et al., 2022). Pollen wall is very sensitive to temperature changes, under HT stress, abnormal exine formation and patterning were observed (Djanaguiraman et al., 2018). Cold can also change the morphology of the exine of pollen, which is thinner at 25/14°C than at 30/20°C (Mercado et al., 1997). In total, two pollen wall-related pathways were detected in the GO enrichment pathway, namely pollen wall assembly (GO:0010208) and the sporopollenin biosynthetic process (GO:0080110). We assume that pollen wall assembly is important for further pollen germination when pollen grains are cultured at a constant temperature of 28°C for 2 days, after which the low temperature stress is lifted. Pollen wall formation is a complex process and the way in which sporopollenins are assembled determines the diversity of the surface morphology of the outer pollen wall. The sporopollenin polymer is the main constituent of exine, and the process of sporopollenin biosynthesis has been extensively studied. To reveal the molecular mechanism of the process of restoration of pollen germination capacity in wild banana, we analysed the GO enrichment of pollen wall-related DEGs. The pollen wall assembly was enriched for three genes whose expression was down-regulated *CYP704B1*, *APY7* (ATP diphosphohydrolases 7), and *β-1,4 - xylosyltransferase*, *CYP704B1* was also down-regulated in the sporopollenin biosynthetic process pathway. In addition, *CYP704B1* was enriched in the KEGG pathway of cutin, suberin and wax biosynthesis and in the pathway of fatty acid degradation. *CYP704B1* is long-chain fatty acid omega-monooxygenase that regulates the conversion of C16 palmitic acid to 16-hydroxyhexadecanoic acid. *cyp704b1* result in impaired pollen walls that lack a normal exine layer (Dobritsa et al., 2009). *CYP704B2*, a homolog of *CYP704B1* in rice, has been reported to catalyse the production of ω-hydroxy fatty acids of the 16- and 18-carbon chains essential for the formation of the cuticle and outer wall of pollen (Li et al., 2010). It has also been shown that *CYP704B1* is significantly upregulated at stage 8 in the male sterile cotton line 1355A, which may lead to excessive accumulation of sporopollenin and thus male sterility (Wu et al., 2015). The possible involvement of *CYP704B1* in the process of sporopollenin biosynthesis, cutin, suberin and wax biosynthesis, fatty acid degradation and pollen wall assembly pathways, as predicted in this study, suggesting the importance of *CYP704B1* in the formation of the wild banana pollen wall. *APY7* is enriched in the pollen wall assembly pathway, and *AtAPY6* and *AtAPY7* transcripts have been reported in *Arabidopsis* to be expressed in mature pollen grains. Both atapy6 and atapy7 knockout mutants alone showed normal pollen development patterns, but when atapy6 and atapy7 were double mutated, their pollen exon patterns were severely defective, with deformed pollen morphology and reduced male fertility (Yang et al., 2013). The *APY7* gene was found to be differentially expressed in the transcriptome before and after pollen germination, but *APY6* did not show differential expression, and we speculate that *APY7* may play a major role in pollen germination in wild type plantain. The β-1, 4-xylosyltransferase is a member of the *GT43* family of glycosyltransferases. Double mutations in β-1, 4 - xylosyltransferase and *IRX14* have been shown to result in loss of secondary fibre wall thickening, which promotes xylan synthesis and is therefore involved in cell wall formation (Lee et al., 2010). Differential expression of these members can ensure the normal assembly process of the pollen wall, which is important for further pollen germination.

In addition, sugar metabolism is important for maintaining the integrity of the pollen wall. We identified members in Amino sugar and nucleotide sugar metabolism and Galactose metabolism that are closely related to cell wall formation, including *UDP-glucose 4, 6-dehydratase* (Ma03_g33030), and *UDP-glucuronate 4-epimerase* (Ma04_g18460). *UDP-glucose 4, 6-dehydratase* have been shown to play a role in maintaining cell wall integrity (Sen et al., 2011). *UDP-glucose 4-epimerase* mediates the interconversion between UDP-Gal, a precursor of monogalactosylceride (MGDG) synthesis in plant cell walls and cell membrane components. UDP-D-glucuronic acid 4-epimerase catalyses the interconversion between uridine diphosphate (UDP-Glc A) and uridine diphosphate-galacturonic acid (UDP-Gal A), which is a key enzyme in the regulation of pectin biosynthesis. It has been reported that ERF2 from lychee plays a role in cell wall metabolism during fruit abscission by directly targeting and inhibiting the expression of UDP-D-glucuronic acid 4-epimerase (Yi et al., 2021). We therefore suggest that the sugar metabolism also plays an important role in the recovery of germination capacity in wild banana. In summary, we conclude that pollen wall remodelling is important for the recovery of germination capacity in wild banana (**Supplementary Tables S4**, **S5**).

### 4.5 MYBs may act as major TFs to target and regulate genes related to pollen development in wild banana

MYB, the largest class of transcription factors in plants, plays an important role in plant growth and development. A number of MYBs have been shown to be involved in pollen growth and development. MYB97, MYB101 and MYB120 participate in pollen tube reception (Liang et al., 2013). Rice CSA2 and CSA regulate sugar transport in anthers under LD and SD conditions, respectively (Wang et al., 2021). AtMYB81 is a specific regulator of microspore development that promotes pollen mitosis I and cell lineage formation (Oh et al., 2020). MS188 is a key regulator of the activation of sporophytic pollen synthesis (Ke et al., 2018). These studies have shown that MYB can activate relevant target genes involved in the process of pollen growth and development. In our study, 14 differentially expressed MYBs targeting 16 genes related to wild banana pollen development were predicted (**Figure 7C**, **Supplementary Tables S7**, **8**). These 16 genes were involved many pollen development pathways (**Supplementary Table S5**). These MYB target genes possess different functions, for example: carboxylesterase 18 (Ma09_g07920) may act on carboxylic esters and are involved in plant metabolic processes and defense responses (Cao et al., 2019). In mammals, carboxylesterases can be involved in lipid metabolism and energy homeostasis (Lian et al., 2018). This is probably the reason for its high expression at 28°C, mediating energy homeostasis by mediating lipid metabolism. AtHMGB15 binds to DNA *in vitro* and interacts with AGL66 and AGL104, required for pollen maturation and pollen tube growth to participate in pollen regulation (Xia et al., 2014). HMG B9 (high mobility group B protein 9-like, Ma06_g22410) was enriched in pollen tube, pollen tube growth, pollen tube growth, pollen tube development, and pollen germinatio in our study, we speculate that it may also play an important role in pollen development; CYP704B1 (Ma08_g18570) was enriched in three pathways, plays an important role in pollen wall formation (Dobritsa et al., 2009). In a word, the MYB may be involved in pollen germination and multiple growth and development processes by targeting these target genes in wild banana.

## 5 Conclusion

In this study, we selected the wild banana pollen that had lost its germination ability under low temperature stress as the control group (CK) and the wild banana pollen that had recovered its germination ability under constant temperature incubation of 28°C for 2 days as the treatment group (T) for transcriptome sequencing. A total of 921 DEGs were detected in CK vs T, of which 265 were up-regulated and 656 were down-regulated. The KEGG pathway is mainly enriched in the metabolism of lipids, sugars and amino acids. TCA cycle and the sesquiterpenoid and triterpenoid biosynthetic pathways were also significantly enriched in the KEGG pathway. And we found that some DEGs may be associated with pollen wall formation and maintenance of DNA methylation levels. The GO pathway was enriched for a total of 24 DEGs related to pollen germination, of which 16 DEGs received targeted regulation by 14 MYBs. Our study suggests that the balance between various metabolic processes, pollen wall integrity, maintenance of DNA methylation, DNA repair and regulation of MYBs are essential for germination of wild banana pollen (**Figures 7**, **8**). This study is instructive in guiding banana breeding, and hybrid selection is a common breeding tool for bananas. In future banana breeding efforts, we can use temperature changes to promote germination of wild banana pollen and improve the fertile nature of the parents. At the same time, the DEGs selected in this study can be used to verify the functions of their genes by transgenic techniques, further resolve the molecular mechanism of wild banana pollen germination. Furthermore, this study shows that metabolism is important for wild banana pollen germination and that metabolome sequencing can be used to identify key metabolites in the pollen germination process and provide more useful information for banana breeding.

## Data availability statement

The datasets presented in this study can be found in online repositories. The names of the repository/repositories and accession number(s) can be found below: NCBI BioProject accession number: PRJNA935075.

## Author contributions

CuZ: Conceptualization, Investigation, Writing – original draft. CeZ: Investigation, Writing – review & editing. XX: Investigation, Writing – review & editing. ML: Investigation, Writing – review & editing. NT: Investigation, Writing – review & editing. ZZ: Writing – review & editing. YC: Writing – review & editing. XH: Writing – review & editing. YL: Supervision, Writing – review & editing. ZL: Conceptualization, Supervision, Writing – review & editing.

## Supplementary material

Supplementary Table 2 The significant enrichment pathways (p < 0.01) in each type of GO enrichment pathway.

Supplementary Table 3 The information of top 20 KEGG pathways.

Supplementary Table 4 The FPKM of DEGs in this study.

Supplementary Table 5 The information of DEGs related to the recovery of pollen germination.

Supplementary Table 6 The TFs likely to be subject to possible regulation of genes associated with the recovery of pollen germination.

Supplementary Table 7 The TFBS enrichment analysis of 14 differentially expressed MYBs.

Supplementary Table 8 Identification of cis-element related to the DEGs related to the recovery of pollen germination.

## References

[B1] Alegre S. Pascual J. Trotta A. Angeleri M. Rahikainen M. Brosche M.. (2020). Evolutionary conservation and post-translational control of S-adenosyl-L-homocysteine hydrolase in land plants. PloS One 15 (7), e0227466. doi: 10.1371/journal.pone.0227466 32678822 PMC7367456

[B2] Ariizumi T. Toriyama K. (2011). Genetic regulation of sporopollenin synthesis and pollen exine development. Annu. Rev. Plant Biol. 62, 437–460. doi: 10.1146/annurev-arplant-042809-112312 21275644

[B3] Armstrong C. T. Anderson J. R. Denton R. M. (2014). Studies on the regulation of the human E1 subunit of the 2-oxoglutarate dehydrogenase complex, including the identification of a novel calcium-binding site. Biochem. J. 459 (2), 369–381. doi: 10.1042/BJ20131664 24495017

[B4] Azman K. Zakaria R. (2019). D-Galactose-induced accelerated aging model: an overview. Biogerontology . 20 (6), 763–782. doi: 10.1007/s10522-019-09837-y 31538262

[B5] Basson C. Groenewald J.-H. Kossmann J. Cronjé C. Bauer R. (2011). Upregulation of pyrophosphate: fructose 6-phosphate 1-phosphotransferase (PFP) activity in strawberry. Transgenic Res. 20 (4), 925–931. doi: 10.1007/s11248-010-9451-0 20960058

[B6] Belužić L. Grbeša I. Belužić R. Park J. H. Kong H. K. Kopjar N.. (2018). Knock-down of *AHCY* and depletion of adenosine induces DNA damage and cell cycle arrest. Sci. Rep. 8 (1), 14012. doi: 10.1038/s41598-018-32356-8 30228286 PMC6143609

[B7] Cao X. Duan W. Wei C. Chen K. Grierson D. Zhang B. (2019). Genome-wide identification and functional analysis of carboxylesterase and methylesterase gene families in peach (Prunus persica L. Batsch). Front. Plant 10, 1511. doi: 10.3389/fpls.2019.01511 PMC6884059 31824538

[B8] Chen L. Yang D. Zhang Y. Wu L. Zhang Y. Ye L.. (2018). Evidence for a specific and critical role of mitogen-activated protein kinase 20 in uni-to-binucleate transition of microgametogenesis in tomato. New Phytol. 219 (1), 176–194. doi: 10.1111/nph.15150 29668051

[B9] Chen B. Zhang Y. Lu Y. Wang J. Zhang S. Lan H.. (2016). DNA methylation analysis of sterile and fertile CMS-C hybrids and their parents in maize. J. Plant Biochem. Biotechnol. 25 (1), 3–11. doi: 10.1007/s13562-015-0298-6

[B10] Condori-Apfata J. Batista-Silva W. Medeiros D. Vargas J. Valenteet L. Heynekeal E.. (2019). The *Arabidopsis* E 1 subunit of the 2-oxoglutarate dehydrogenase complex modulates plant growth and seed production. Plant Mol. Biol. 101, 183–202. doi: 10.1007/s11103-019-00900-3 31286324

[B11] Djanaguiraman M. Perumal R. Jagadish S. V. K. Ciampitti I. A. Welti R. Prasad P. V. V. (2018). Sensitivity of sorghum pollen and pistil to high-temperature stress. Plant Cell Environment. 41, 1065–1082. doi: 10.1111/pce.13089 PMC5904002 29044571

[B12] Dobritsa A. A. Shrestha J. Morant M. Pinot F. Matsuno M. Swanson R.. (2009). *CYP704B1* is a long-chain fatty acid ω-hydroxylase essential for sporopollenin synthesis in pollen of *Arabidopsis* . Plant Physiol. 151 (2):574–589. doi: 10.1104/pp.109.144469 PMC2754625 19700560

[B13] Doughty J. Wong Y. Dickinson H. (2000). Cysteine-rich pollen coat proteins (pcps) and their interactions with stigmatic s (incompatibility) and s-related proteins in brassica: putative roles in si and pollination. Ann. Bot. 85 (1), 161–169. doi: 10.1006/anbo.1999.1031

[B14] Furuyama T. Dzelzkalns V. A. (1999). A novel calcium-binding protein is expressed in Brassica pistils and anthers late in flower development. Plant Mol. Biol. 39 (4), 729–737. doi: 10.1023/A:1006169808171 10350087

[B15] Gehring M. Reik W. Henikoff S. (2009). DNA demethylation by DNA repair. Trends Genet. 25 (2), 82–90. doi: 10.1016/j.tig.2008.12.001 19144439

[B16] Ha N. T. Lee C. H. (2020). Roles of farnesyl-diphosphate farnesyltransferase 1 in tumour and tumour microenvironments. Cells 9 (11), 2352. doi: 10.3390/cells9112352 33113804 PMC7693003

[B17] Han Y. Hu M. Ma X. Yan G. Wang C. Jiang S.. (2022). Exploring key developmental phases and phase-specific genes across the entirety of anther development in maize. J. Integr. Plant Biol. 64 (7), 1394–1410. doi: 10.1111/jipb.13276 35607822 PMC10360140

[B18] Hirsche J. Fernández J. M. G. Stabentheiner E. Großkinsky D. K. Roitsch T. (2017). Differential effects of carbohydrates on *arabidopsis* pollen germination. Plant Cell Physiol. 58 (4), 691–201. doi: 10.1093/pcp/pcx020 28339807

[B19] Hu L. Liang W. Yin C. Cui X. Zong J. Wang X.. (2011). Rice *MADS3* regulates ROS homeostasis during late anther development. Plant Cell 23, 515–533. doi: 10.1105/tpc.110.074369 21297036 PMC3077785

[B20] Jessen D. Olbrich A. Knüfer J. Krüger A. Hoppert M. Polle A.. (2011). Combined activity of *LACS1* and *LACS4* is required for proper pollen coat formation in *Arabidopsis* . Plant J. 68 (4), 715–726. doi: 10.1111/j.1365-313X.2011.04722.x 21790813

[B21] Jiang Y. Lahlali R. Karunakaran C. Kumar S. Davis A. R. (2015). Seed set, pollen morphology and pollen surface composition response to heat stress in field pea. Plant Cell Environ. 38 (11), 2387–2397. doi: 10.1111/pce.12589 26081983

[B22] Ke W. Guo Z. L. Zhou W. T. Cheng Z. Zhang Z. Y. Yue L.. (2018). The regulation of sporopollenin biosynthesis genes for rapid pollen wall formation. Plant Physiol. 178, 283. doi: 10.1104/pp.18.00219 30018171 PMC6130021

[B23] Khan A. H. Ma Y. Wu Y. Akbar A. Shaban M. Ullah A.. (2023). High-temperature stress suppresses allene oxide cyclase 2 and causes male sterility in cotton by disrupting jasmonic acid signaling. Crop J. 1 (11), 33–45. doi: 10.1016/j.cj.2022.05.009

[B24] Khouider S. Borges F. LeBlanc C. Ungru A. Schnittger A. Martienssen R.. (2021). Male fertility in *Arabidopsis* requires active DNA demethylation of genes that control pollen tube function. Nat. Commun. 18, 12(1):410. doi: 10.1038/s41467-020-20606-1 PMC7813888 33462227

[B25] Kiran A. Sharma P. Awasthi R. Nayyar H. Seth R. Chandel S. S.. (2021). Disruption of carbohydrate and proline metabolism in anthers under low temperature causes pollen sterility in chickpea. Environ. Exp. Bot. 188, 104500. doi: 10.1016/j.envexpbot.2021.104500

[B26] Kong X. Khan A. Li B. Zheng J. Dawar F. U. Li M.. (2020). Deviant DNA methylation play a key role in the pollen abortion of Gossypium barbadense L. cytoplasmic male sterility. Ind. Crops Prod. 154, 112622. doi: 10.1016/j.indcrop.2020.112622

[B27] Krawczyk H. E. Rotsch A. H. Herrfurth C. Scholz P. Shomroni O. Salinas-Riester G.. (2022). Heat stress leads to rapid lipid remodeling and transcriptional adaptations in Nicotiana tabacum pollen tubes. Plant Physiol. 189 (2), 490–515. doi: 10.1093/plphys/kiac127 35302599 PMC9157110

[B28] Lee C. Teng Q. Huang W. Zhong R. Ye Z. H. (2010). The *Arabidopsis* family *GT43* glycosyltransferases form two functionally nonredundant groups essential for the elongation of glucuronoxylan backbone. Plant Physiol. 153 (2), 526–541. doi: 10.1104/pp.110.155309 20335400 PMC2879797

[B29] Li H. Pinot F. Sauveplane V. Werck-Reichhart D. Diehl P. Schreiber L.. (2010). Cytochrome P450 family member *CYP704B2* catalyzes the -hydroxylation of fatty acids and is required for anther cutin biosynthesis. Plant Cell 22 (1), 173–190. doi: 10.1105/tpc.109.070326 20086189 PMC2828706

[B30] Li C. Tao R.-F. Li Y. Duan M.-H. Xu J.-H. (2020). Transcriptome analysis of the thermosensitive genic male-sterile line provides new insights into fertility alteration in rice (Oryza sativa). Genomics 112 (3), 2119–2129. doi: 10.1016/j.ygeno.2019.12.006 31837402

[B31] Li H. Tiwari M. Tang Y. Wang L. Yang S. Long H.. (2022). Metabolomic and transcriptomic analyses reveal that sucrose synthase regulates maize pollen viability under heat and drought stress. Ecotoxicol. Environ. Saf. 246, 114191. doi: 10.1016/j.ecoenv.2022.114191 36265405

[B32] Li J. Wang C. Liang W. Zhang J. Jiang C. K. Liu Y.. (2023). Functional importance and divergence of plant apurinic/apyrimidinic endonucleases in somatic and meiotic DNA repair. Plant Cell. 28, koad056. doi: 10.1093/plcell/koad056 PMC10226563 36856605

[B33] Lian J. Nelson R. Lehner R. (2018). Carboxylesterases in lipid metabolism: from mouse to human. Protein Cell. 9 (2), 178–195. doi: 10.1007/s13238-017-0437-z 28677105 PMC5818367

[B34] Liang Y. Tan Z. M. Zhu L. Niu Q. K. Zhou J. J. Li M.. (2013). MYB97, MYB101 and MYB120 function as male factors that control pollen tube-synergid interaction in *arabidopsis thaliana* fertilization. PloS Genet. 9 (11), e1003933. doi: 10.1371/journal.pgen.1003933 24278028 PMC3836714

[B35] Lin Y. Lai Z. (2010). Reference gene selection for qPCR analysis during somatic embryogenesis in longan tree. Plant Sci. 178, 359–365. doi: 10.1016/j.plantsci.2010.02.005

[B36] Liu W. Cheng C. Chen F. Ni S. Lin Y. Lai Z. (2018). High-throughput sequencing of small RNAs revealed the diversified cold-responsive pathways during cold stress in the wild banana (Musa itinerans). BMC Plant Biol. 18 (1), 1–26. doi: 10.1186/s12870-018-1483-2 30486778 PMC6263057

[B37] Liu S. Li Z. Wu S. Wan X. (2021). The essential roles of sugar metabolism for pollen development and male fertility in plants. Crop J. 9 (6), 14. doi: 10.1016/j.cj.2021.08.003

[B38] Ma Y. Min L. Wang J. Li Y. Wu Y. Hu Q.. (2021). A combination of genome-wide and transcriptome-wide association studies reveals genetic elements leading to male sterility during high temperature stress in cotton. New Phytol. 231 (1), 165–181. doi: 10.1111/nph.17325 33665819 PMC8252431

[B39] Maksimov M. (2018). The role of reactive oxygen species in pollen germination in Picea pungens (blue spruce). Plant Reprod. 31 (4), 357–365. doi: 10.1007/s00497-018-0335-4 29619606

[B40] Mamun E. A. Cantrill L. C. Overall R. L. Sutton B. G. (2013). Mechanism of low-temperature-induced pollen failure in rice. Cell Biol. Int. 34 (5), 469–476. doi: 10.1042/CBI20090417 20100170

[B41] Meng D. He M. Bai Y. Xu H. Dandekar A. M. Fei Z.. (2018). Decreased sorbitol synthesis leads to abnormal stamen development and reduced pollen tube growth *via* an MYB transcription factor, MdMYB39L, in apple (Malus domestica). New Phytol. 217 (2), 641–656. doi: 10.1111/nph.14824 29027668

[B42] Mercado J. A. Mar Trigo M. Reid M. S. Valpuesta V. Quesada M. A. (1997). Effects of low temperature on pepper pollen morphology and fertility: Evidence of cold induced exine alterations. J. Pomol. Hortic. Sci. 72 (2), 317–326. doi: 10.1080/14620316.1997.11515518

[B43] Mortusewicz O. Schermelleh L. Walter J. Cardoso M. C. Leonhardt H. (2005). Recruitment of DNA methyltransferase I to DNA repair sites. Proc. Natl. Acad. Sci. U S A. 102 (25), 8905–8909. doi: 10.1073/pnas.0501034102 15956212 PMC1157029

[B44] Muhlemann J. K. Younts T. L. Muday G. K. (2018). Flavonols control pollen tube growth and integrity by regulating ROS homeostasis during high-temperature stress. Proc. Natl. Acad. Sci. 115 (47), E11188–E11197. doi: 10.1073/pnas.1811492115 30413622 PMC6255205

[B45] Nelms B. Walbot V. (2022). Gametophyte genome activation occurs at pollen mitosis I in maize. Science 375 (6579), 424–429. doi: 10.1126/science.abl7392 35084965

[B46] Ni S. Lin Z. Liu Y. Chen Y. Tian X. Wang X.. (2021). Genome-wide identification of banana maSULTR3 family members, cloning of maSULTR3.1-2 gene and analysis of expression patterns. J. Trop. Crops 42 (3), 12. doi: 10.3969/j.issn.1000-2561.2021.03.004

[B47] Oh S.-A. Nguyen T. Hoai T. Park H.-J. Zhao M. Twell D.. (2020). MYB81, a microspore-specific GAMYB transcription factor, promotes pollen mitosis I and cell lineage formation in *Arabidopsis* . Plant J. 101 (3), 590–603. doi: 10.1111/tpj.14564 31610057

[B48] Okusaka K. Hiratsuka S. (2009). Fructose inhibits pear pollen germination on agar medium without loss of viability. Sci. Hortic. 122 (1), 51–55. doi: 10.1016/j.scienta.2009.03.024

[B49] Pasqualini S. Cresti M. Casino C. Faleri C. Frenguelli G. Tedeschini E.. (2015). Roles for NO and ROS signalling in pollen germination and pollen-tube elongation in Cupressus arizonica. Biol. Plant. 59 (4), 735–744. doi: 10.1007/s10535-015-0538-6

[B50] Pillay M. Tripathi L. (2007). "Banana breeding," in *Breeding major food staples*. Eds. M. S. Kang and P. M. Priyadarshan (Boston, USA: Blackwell Publishing), 393–428.

[B51] Qi Z.-Y. Wang K.-X. Yan M.-Y. Kanwar M. K. Li D.-Y. Wijaya L.. (2018). Melatonin alleviates high temperature-induced pollen abortion in Solanum lycopersicum. Molecules 23 (2), 386. doi: 10.3390/molecules23020386 29439470 PMC6017144

[B52] Rieu I. Twell D. Firon N. (2017). Pollen development at high temperature: from acclimation to collapse. Plant Physiol. 173 (4), 1967–1976. doi: 10.1104/pp.16.01644 28246296 PMC5373052

[B53] Sen M. Shah B. Rakshit S. Singh V. Sadhale P. P. (2011). UDP-glucose 4, 6-dehydratase activity plays an important role in maintaining cell wall integrity and virulence of candida albicans. PloS Pathog. 7 (11), e1002384. doi: 10.1371/journal.ppat.1002384 22114559 PMC3219719

[B54] Soares T. L. Souza E. Costa M.A.P. d. C. Silva S. Serejo J.A. d. S. (2014). *In vivo* fertilization of banana. Ciencia Rural 44, 37–42. doi: 10.1590/S0103-84782013005000146

[B55] Tian Q. Lu C. Li X. Fang X. (2015). Low temperature treatments of rice (*Oryza sativa* L.) anthers changes polysaccharide and protein composition of the anther walls and increases pollen fertility and callus induction. Plant Cell Tissue Organ Cult. (PCTOC) 120 (1), 89–98. doi: 10.1007/s11240-014-0582-5

[B56] Wang Z. Baulcombe D. C. (2020). Transposon age and non-CG methylation. Nat. Commun. 11 (1), 1221. doi: 10.1038/s41467-020-14995-6 32144266 PMC7060349

[B57] Wang D. Li J. Sun L. Hu Y. Yu J. Wang C.. (2021). Two rice MYB transcription factors maintain male fertility in response to photoperiod by modulating sugar partitioning. New Phytol. 231 (4), 1612–1629. doi: 10.1111/nph.17512 34031889

[B58] Wang J. Yu Y.-C. Li Y. Chen L.-Q. (2022). Hexose transporter *SWEET5* confers galactose sensitivity to *Arabidopsis* pollen germination *via* a galactokinase. Plant Physiol. 189 (1), 388–401. doi: 10.1093/plphys/kiac068 35188197 PMC9070816

[B59] Wei C. Pingping G. Jingzhe G. Hui L. Ruizi L. Weiman X.. (2018). Glycolysis regulates pollen tube polarity *via* Rho GTPase signaling. PloS Genet. 14 (4), e1007373. doi: 10.1371/journal.pgen.1007373 29702701 PMC5942846

[B60] Wu Y. Min L. Wu Z. Yang L. Zhu L. Yang X.. (2015). Defective pollen wall contributes to male sterility in the male sterile line 1355A of cotton. Sci. Rep. 5, 9608. doi: 10.1038/srep09608 26043720 PMC4456728

[B61] Xia C. Wang Y. J. Liang Y. Niu Q. K. Tan X. Y. Chu L. C.. (2014). The ARID-HMG DNA-binding protein AtHMGB15 is required for pollen tube growth in *Arabidopsis thaliana* . Plant J. 79 (5), 741–756. doi: 10.1111/tpj.12582 24923357

[B62] Xie H. T. Wan Z. Y. Li S. Zhang Y. (2014). Spatiotemporal production of reactive oxygen species by NADPH oxidase is critical for tapetal programmed cell death and pollen development in *Arabidopsis* . Plant Cell 26, 2007–2023. doi: 10.1105/tpc.114.125427 24808050 PMC4079365

[B63] Xie D. Zheng X. Zhou C. Kanwar M. Zhou J. (2022). Functions of redox signaling in pollen development and stress response. Antioxidants 11, 287. doi: 10.3390/antiox11020287 35204170 PMC8868224

[B64] Yang J. Wu J. Romanovicz D. Clark G. Roux S. J. (2013). Co-regulation of exine wall patterning, pollen fertility and anther dehiscence by *Arabidopsis apyrases 6* and *7* . Plant Physiol. Biochem. 69, 62–73. doi: 10.1016/j.plaphy.2013.04.022 23728389

[B65] Yi J. Moon S. Lee Y. S. Zhu L. Liang W. Zhang D.. (2016). Defective Tapetum Cell Death 1 (DTC1) regulates ROS levels by binding to metallothionein during tapetum degeneration. Plant Physiol. 170, 1611–1623. doi: 10.1104/pp.15.01561 26697896 PMC4775127

[B66] Yi J.-W. Wang Y. Ma X.-S. Zhang J.-Q. Zhao M.-L. Huang X.-M.. (2021). LcERF2 modulates cell wall metabolism by directly targeting a UDP-glucose-4-epimerase gene to regulate pedicel development and fruit abscission of litchi. Plant J. 106 (3), 801–816. doi: 10.1111/tpj.15201 33595139

[B67] Yu J. Han J. Kim Y.-J. Song M. Yang Z. He Y.. (2017). Two rice receptor-like kinases maintain male fertility under changing temperatures. Proc. Natl. Acad. Sci. 114 (46), 12327–12332. doi: 10.1073/pnas.1705189114 29087306 PMC5699033

[B68] Yuan G. Zou T. He Z. Xiao Q. Li G. Liu S.. (2022). *SWOLLEN TAPETUM AND STERILITY 1* is required for tapetum degeneration and pollen wall formation in rice. Plant Physiol. 190 (1), 352–370. doi: 10.1093/plphys/kiac307 35748750 PMC9434214

[B69] Zhang Y. Zhen L. Tan X. Li L. Wang X. (2014). The involvement of hexokinase in the coordinated regulation of glucose and gibberellin on cell wall invertase and sucrose synthesis in grape berry. Mol. Biol. Rep. 41 (12), 7899–7910. doi: 10.1007/s11033-014-3683-7 25163631

[B70] Zhao Q. Zhou L. Liu J. Cao Z. Du X. Huang F.. (2018). Involvement of CAT in the detoxification of HT-induced ROS burst in rice anther and its relation to pollen fertility. Plant Cell Rep. 37 (5), 741–757. doi: 10.1007/s00299-018-2264-y 29464319

