# Supplementary material for: Transcriptome analysis provides insight into the regulatory mechanisms underlying pollen germination recovery at normal high ambient temperature in wild banana (Musa itinerans)
Source: Front Plant Sci. 2023 Sep 26;14:1255418. doi: 10.3389/fpls.2023.1255418 (PMC10562711; doi:10.3389/fpls.2023.1255418)
Supplement: Supplementary file 1 [file DataSheet_1.docx]

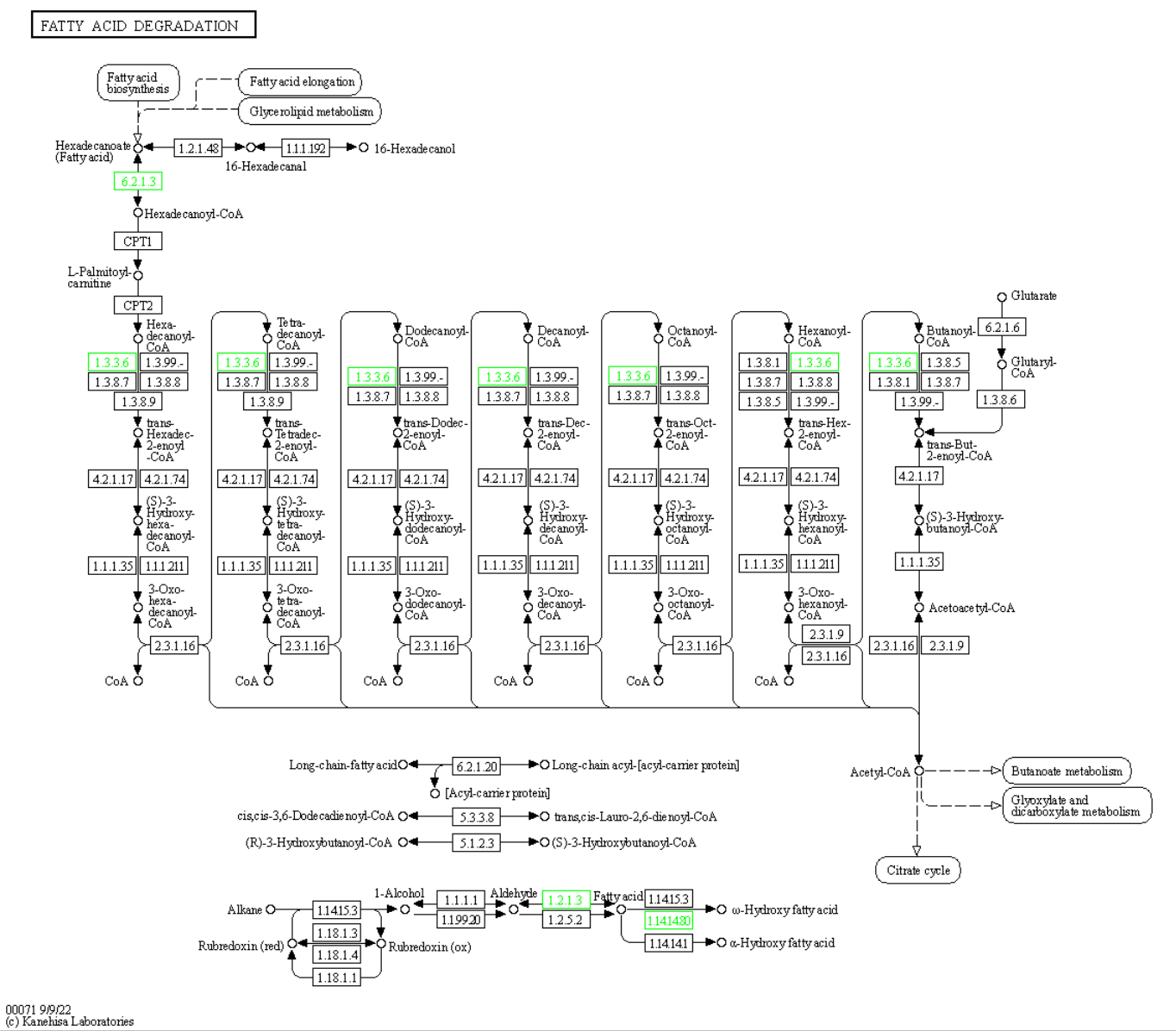


**Supplementary Figure S1:** The KEGG pathway of Fatty acid degradation. Nodes marked in green indicate downregulated genes of experimental group.


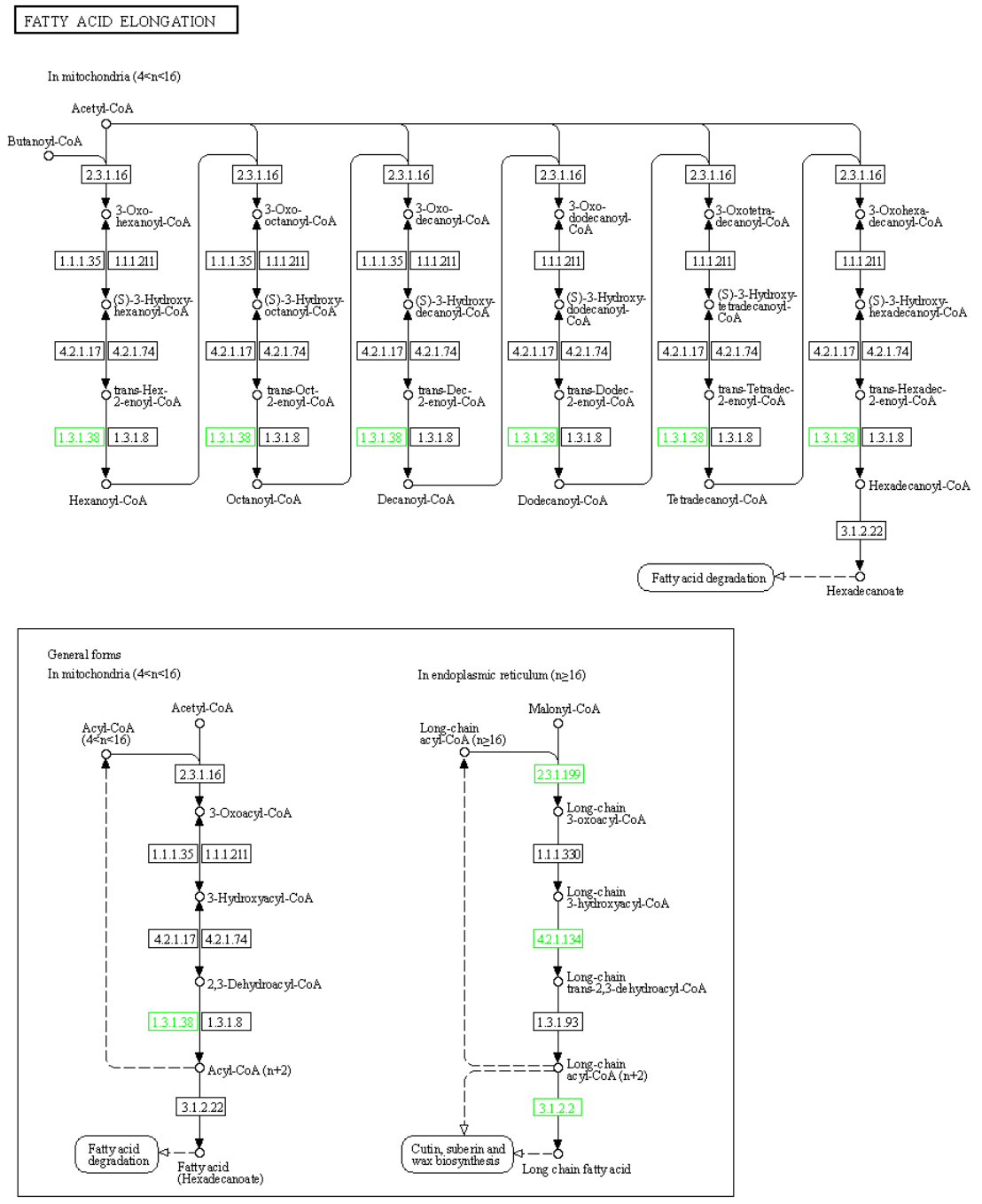


**Supplementary Figure S2:** The KEGG pathway of Fatty acid elongation. Nodes marked in green indicate downregulated genes of experimental group.


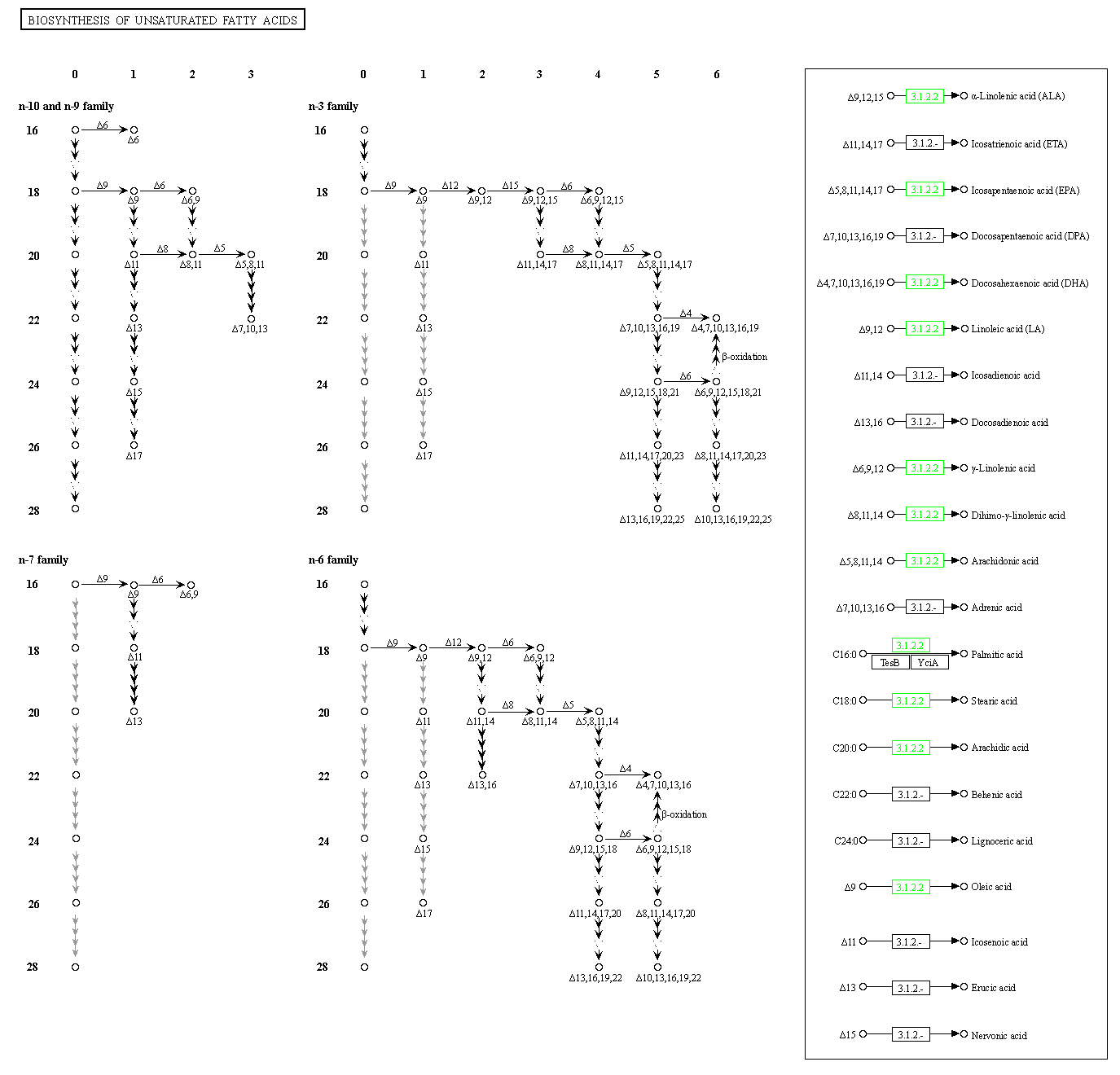


**Supplementary Figure S3:** The KEGG pathway of Biosynthesis of unsaturated fatty acids. Nodes marked in green indicate downregulated genes of experimental group.


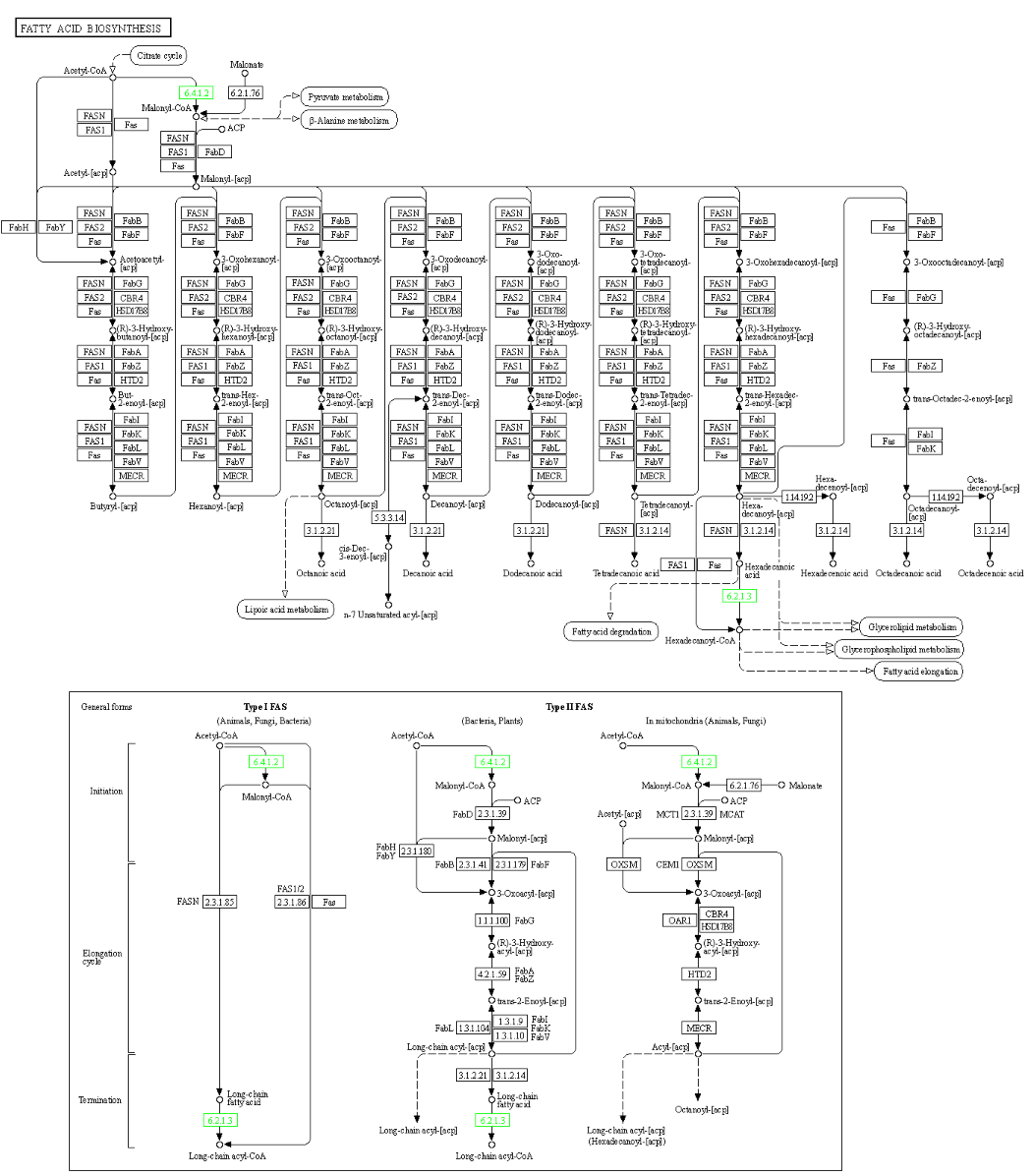


**Supplementary Figure S4:** The KEGG pathway of Fatty acid biosynthesis. Nodes marked in green indicate downregulated genes of experimental group.


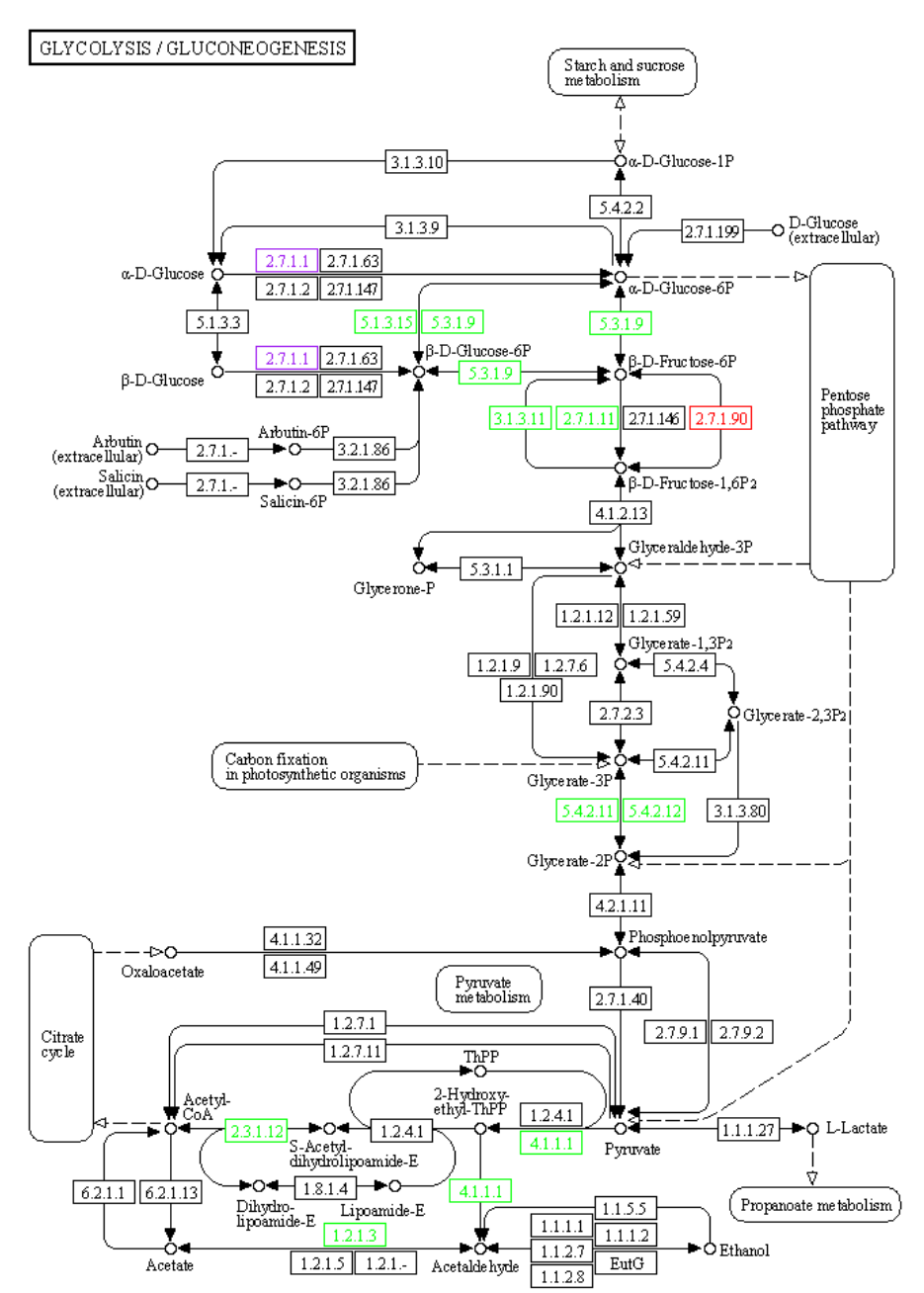


**Supplementary Figure S5:** The KEGG pathway of Glycolysis / Gluconeogenesis. Nodes marked in red indicate upregulated genes of experimental group; nodes marked in green indicate downregulated genes of experimental group; nodes marked in purple indicate both upregulated genes of experimental and control groups.


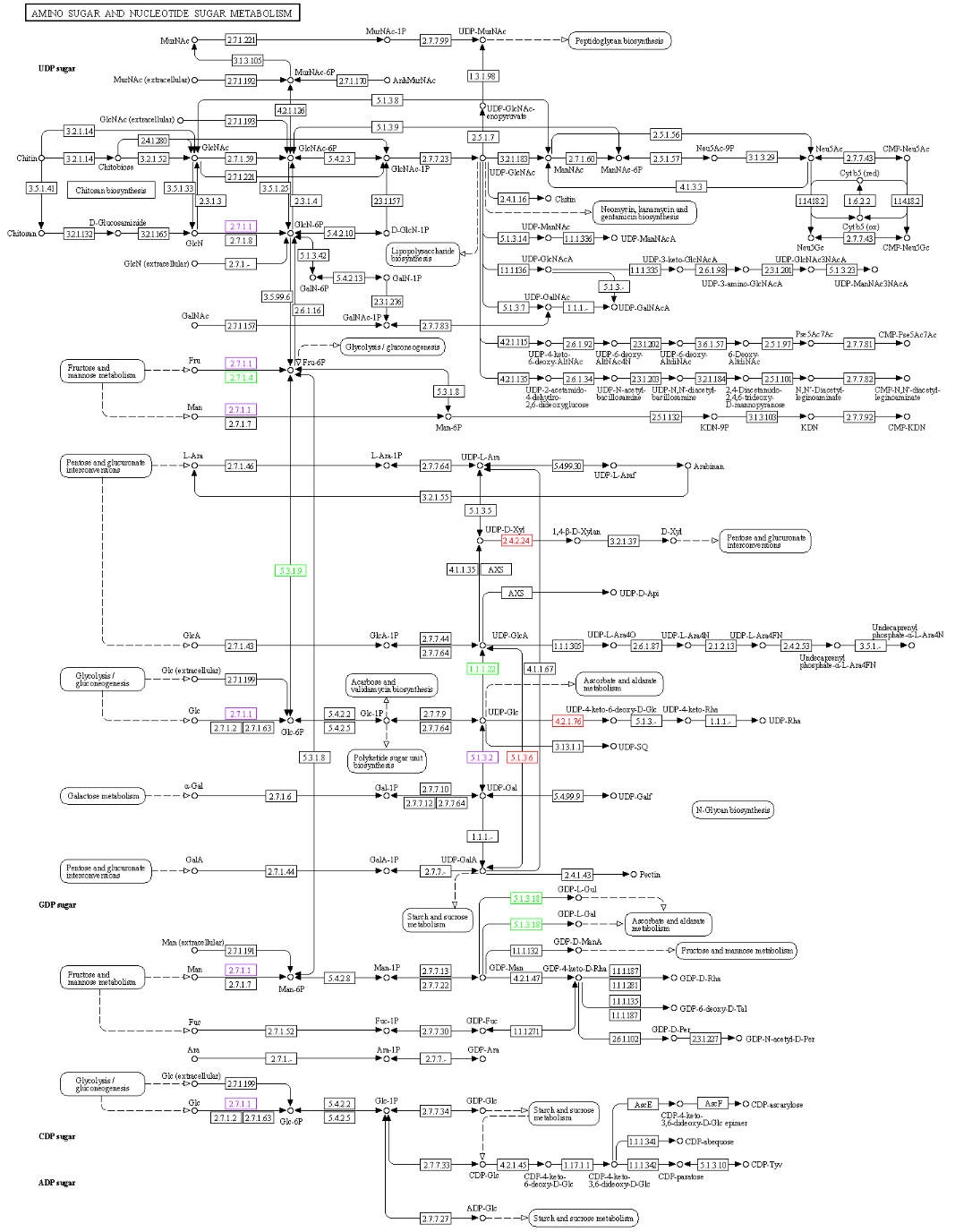


**Supplementary Figure S6:** The KEGG pathway of Amino sugar and nucleotide sugar metabolism. Nodes marked in red indicate upregulated genes of experimental group; nodes marked in green indicate downregulated genes of experimental group; nodes marked in purple indicate both upregulated genes of experimental and control groups.


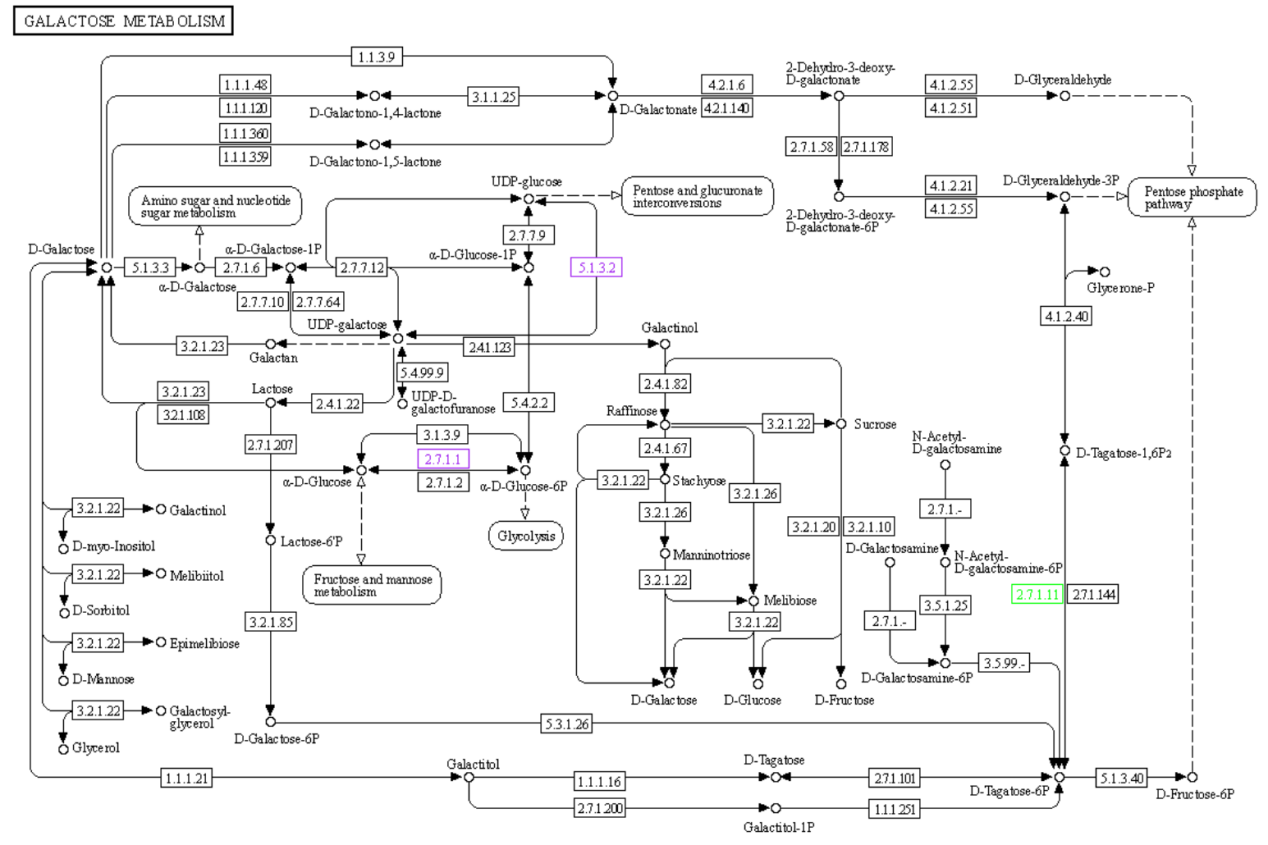


**Supplementary Figure S7:** The KEGG pathway of Galactose metabolism. Nodes marked in green indicate downregulated genes of experimental group; nodes marked in purple indicate both upregulated genes of experimental and control groups.


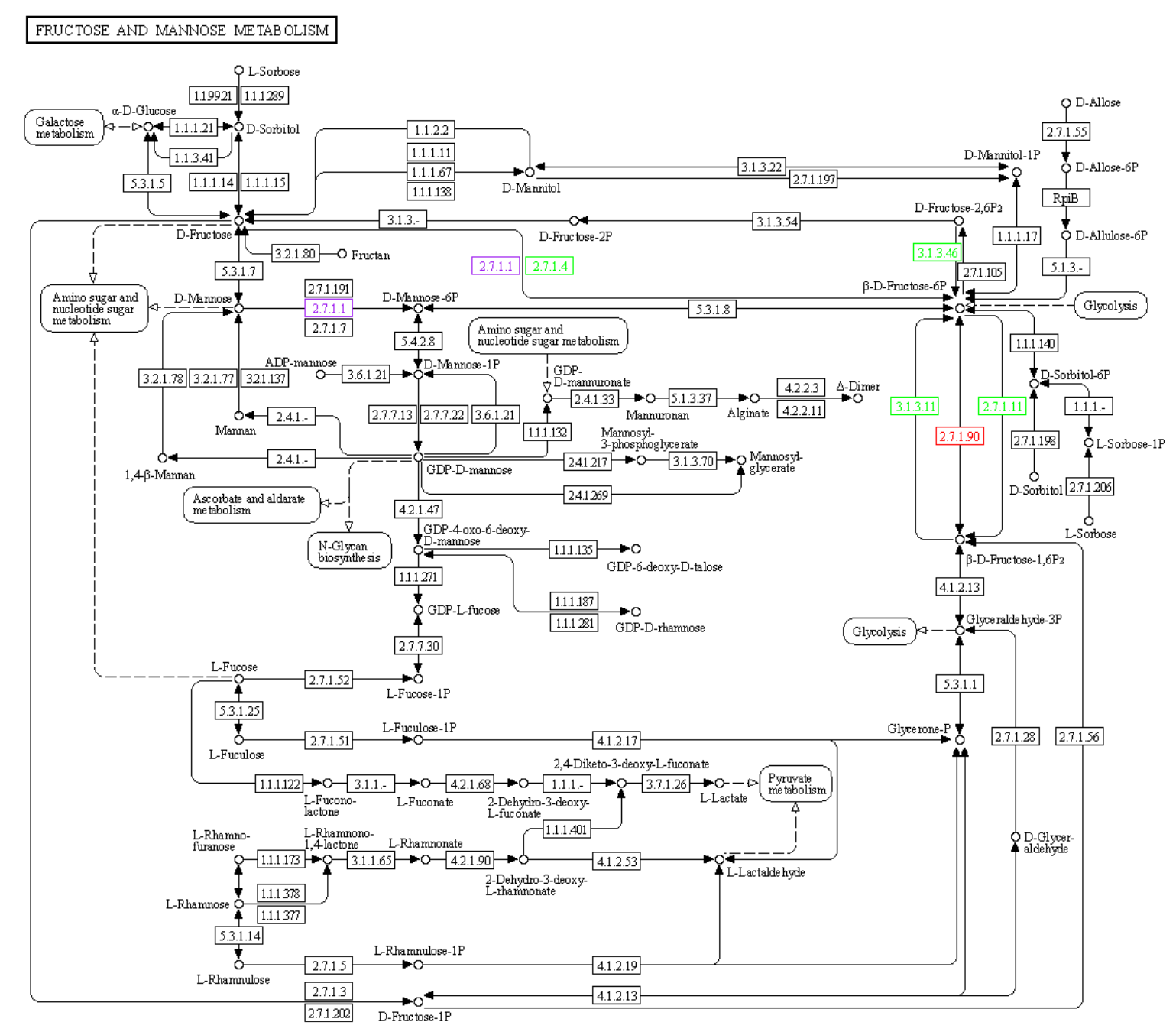


**Supplementary Figure S8:** The KEGG pathway of Fructose and mannose metabolism. Nodes marked in red indicate upregulated genes of experimental group; nodes marked in green indicate downregulated genes of experimental group; nodes marked in purple indicate both upregulated genes of experimental and control groups.


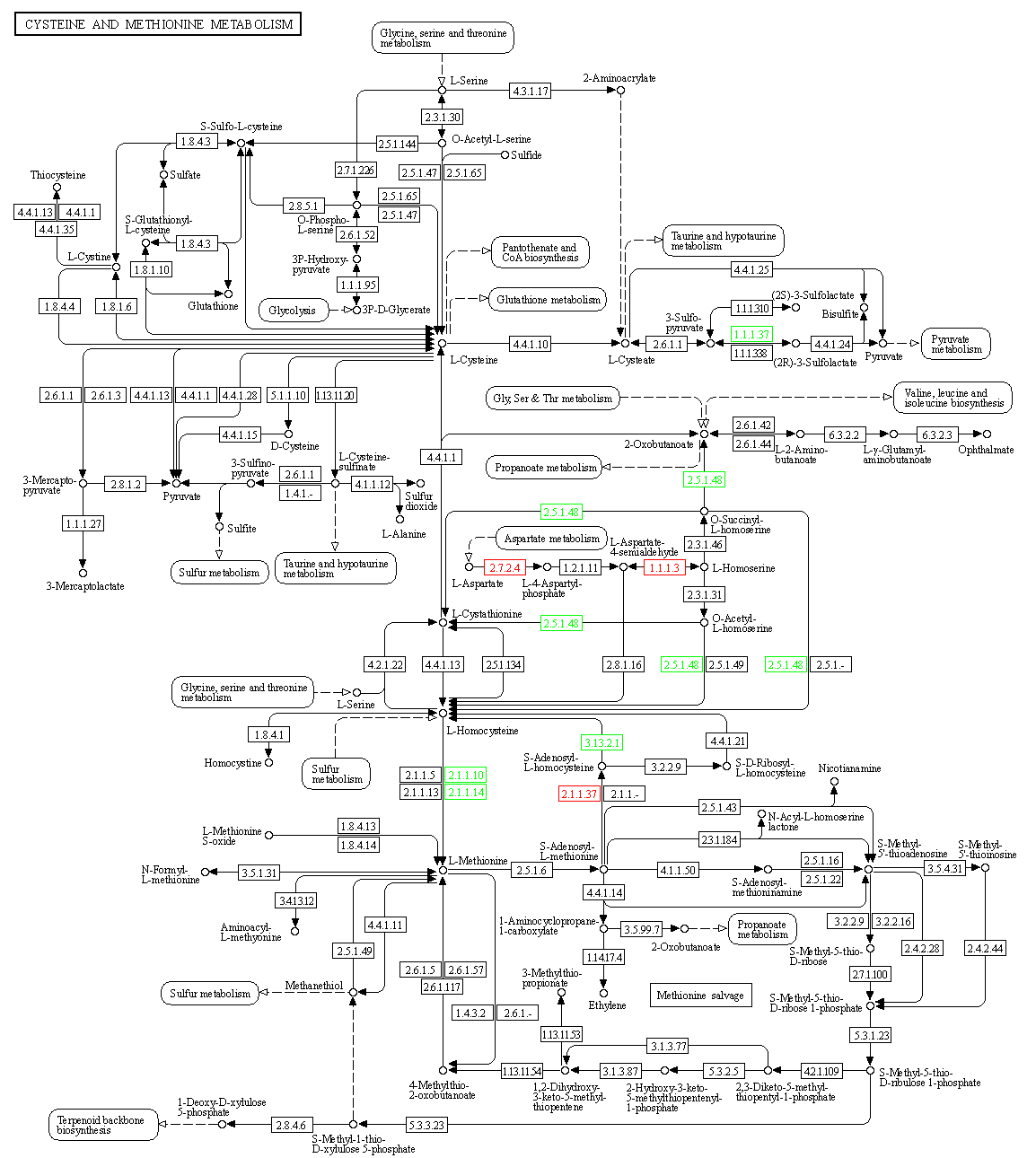


**Supplementary Figure S9:** The KEGG pathway of Cysteine and methionine metabolism. Nodes marked in red indicate upregulated genes of experimental group; nodes marked in green indicate downregulated genes of experimental group.


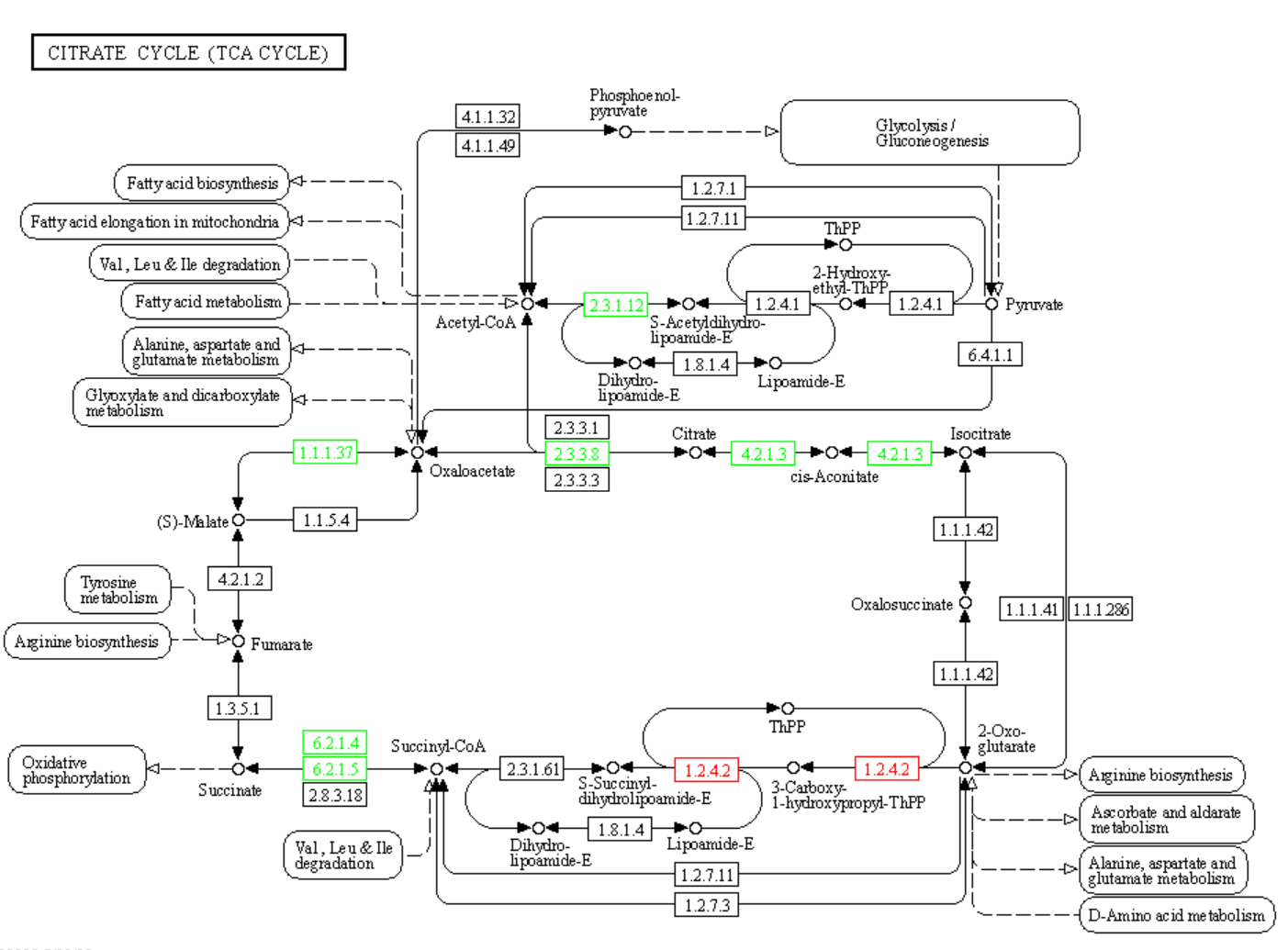


**Supplementary Figure S10:** The KEGG pathway of Citrate cycle (TCA cycle). Nodes marked in red indicate upregulated genes of experimental group; nodes marked in green indicate downregulated genes of experimental group.
